# The Formaldehyde Dehydrogenase SsFdh1 Is Regulated by and Functionally Cooperates with the GATA Transcription Factor SsNsd1 in Sclerotinia sclerotiorum

**DOI:** 10.1128/mSystems.00397-19

**Published:** 2019-09-10

**Authors:** Genglin Zhu, Gang Yu, Xianghui Zhang, Jinliang Liu, Yanhua Zhang, Jeffrey A. Rollins, Jingtao Li, Hongyu Pan

**Affiliations:** a College of Plant Sciences, Jilin University, Changchun, China; b Key Lab of Integrated Crop Pest Management of Shandong Province, College of Plant Health and Medicine, Qingdao Agricultural University, Qingdao, China; c Department of Plant Pathology, University of Florida, Gainesville, Florida, USA; Princeton University

**Keywords:** *Sclerotinia sclerotiorum*, GATA transcription factor, glutathione-dependent formaldehyde dehydrogenase, metabolism, pathogenicity

## Abstract

GATA transcription factors (TFs) are common eukaryotic regulators, and glutathione-dependent formaldehyde dehydrogenases (GD-FDH) are ubiquitous enzymes with formaldehyde detoxification activity. In this study, the formaldehyde dehydrogenase Sclerotinia sclerotiorum Fdh1 (SsFdh1) was first characterized as an interacting partner of a GATA TF, SsNsd1, in S. sclerotiorum. Genetic analysis reveals that SsFdh1 functions in formaldehyde detoxification, nitrogen metabolism, sclerotium development, and pathogenicity. Both SsNsd1 and SsFdh1 harbor typical zinc finger motifs with conserved cysteine residues. SsNsd1 regulates SsFdh1 in two distinct manners. SsNsd1 directly binds to GATA-box DNA in the promoter region of *Ssfdh1*; SsNsd1 associates with SsFdh1 through disulfide bonds formed by conserved Cys residues. The SsNsd1-SsFdh1 interaction and nuclear translocation were found to prevent efficient binding of SsNsd1 to GATA-box DNA. Site-directed point mutation of these Cys residues influences the SsNsd1-SsFdh1 interaction and SsNsd1 DNA binding capacity. SsFdh1 is regulated by and functions jointly with the SsNsd1 factor, providing new insights into the complex transcriptional regulatory mechanisms of GATA factors.

**IMPORTANCE** S. sclerotiorum is a pathogenic fungus with sclerotium and infection cushion development, making S. sclerotiorum one of the most challenging agricultural pathogens with no effective control method. We identified important sclerotium and compound appressorium formation determinants, SsNsd1 and SsFdh1, and investigated their regulatory mechanism at the molecular level. SsNsd1 and SsFdh1 are zinc finger motif-containing proteins and associate with each other in the nucleus. On other hand, SsNsd1, as a GATA transcription factor, directly binds to GATA-box DNA in the promoter region of *Ssfdh1*. The SsNsd1-SsFdh1 interaction and nuclear translocation were found to prevent efficient binding of SsNsd1 to GATA-box DNA. Our results provide insights into the role of the GATA transcription factor and its regulation of formaldehyde dehydrogenase in stress resistance, fungal sclerotium and compound appressorium development, and pathogenicity.

## Abstract (precis)

S. sclerotiorum is a pathogenic fungus with sclerotium and infection cushion development, making S. sclerotiorum one of the most challenging agricultural pathogens with no effective control method. We identified important sclerotium and compound appressorium formation determinants, SsNsd1 and SsFdh1, and investigated their regulatory mechanism at the molecular level. SsNsd1 and SsFdh1 are zinc finger motif-containing proteins and associate with each other in the nucleus. On other hand, SsNsd1, as a GATA transcription factor, directly binds to GATA-box DNA in the promoter region of *Ssfdh1*. The SsNsd1-SsFdh1 interaction and nuclear translocation were found to prevent efficient binding of SsNsd1 to GATA-box DNA. Our results provide insights into the role of the GATA transcription factor and its regulation of formaldehyde dehydrogenase in stress resistance, fungal sclerotium and compound appressorium development, and pathogenicity.

## INTRODUCTION

Sclerotinia sclerotiorum (Lib.) de Bary is a plant-pathogenic filamentous fungus that damages agricultural crops (1, 2). Vegetative hypha of S. sclerotiorum could either form hardened, multicellular sclerotia with viability for several years (3) or develop into a specialized structure called a compound appressorium (infection cushion), which can directly infect host tissue (4). The development of the sclerotia and appressoria makes S. sclerotiorum one of the most challenging agricultural pathogens to control (5, 6).

The GATA-type transcription factors (TF) commonly occur in fungi, plants, and metazoans, with a DNA-binding motif, usually constituted by a four-cysteine zinc finger (ZnF), specifically binding to a six-base-pair (A/T)GATA(A/G) DNA sequence (7, 8). As transcriptional activators or repressors, the regulatory roles of GATA TFs are diverse in controlling the expression of downstream genes and governing cell differentiation and development. In fungi, GATA TFs play key roles in nitrogen metabolism, light perception, siderophore biosynthesis, and mating-type switching (9–11). In S. sclerotiorum, there are nine GATA-type TFs (12). We studied one of these GATA-type TFs, S. sclerotiorum Nsd1 (SsNsd1), orthologous to Aspergillus nidulans nsdD (for “never in sexual development D”), and found that SsNsd1 is functionally involved in asexual-sexual development, sclerotium development, and compound appressorium formation (6). However, there is little information on the SsNsd1-mediated signal pathways, its interacting partners, and its role in nitrogen metabolism in S. sclerotiorum.

Glutathione-dependent formaldehyde dehydrogenases (GD-FDH; EC 1.2.1.1) are a ubiquitous class of enzymes, found in both prokaryotes and eukaryotes. They are orthologous to S-(hydroxymethyl) glutathione dehydrogenases (SFA; EC 1.1.1.284) in yeast (13, 14). The FDHs or SFAs belong to the zinc-containing alcohol dehydrogenase (ADH) family, with a primary function in biological systems detoxifying endogenous and exogenous formaldehyde (15, 16). The FDHs are also known to partially regulate nitric oxide (NO) metabolism through specifically catalyzing the reduction of S-nitrosoglutathione (GSNO) (14, 15, 17). In Cryptococcus neoformans, GSNO reductase with NO-consuming activity contributes to defense against the nitrosative stress mounted by the host and promotes fungal virulence (18). Magnaporthe oryzae SFA1 (MoSFA1)-mediated NO metabolism is important for redox homeostasis, conidiation, and virulence (14). However, the presence and functions of FDH in S. sclerotiorum remain unclear.

To study the SsNsd1-mediated regulatory pathway, we used a yeast two-hybrid (Y2H) method to identify its interacting proteins and identified a formaldehyde dehydrogenase (SS1G_10135), SsFdh1, in S. sclerotiorum. The functions of SsFdh1 were characterized by a gene knockout strategy with a focus on nitrogen metabolism, sclerotium development, and virulence on host plants. We further characterized the importance of key Cys residues in the ZnF motifs for SsNsd1-SsFdh1 interaction, SsNsd1 GATA-box DNA binding, and protein functions. This information reveals the biological functions of SsFdh1 and SsNsd1-associated regulatory pathways for nitrogen metabolism and development in S. sclerotiorum.

## RESULTS

### SsFdh1 functions in formaldehyde detoxification and sodium nitroprusside (SNP) tolerance.

We previously reported that *Ssnsd1* was involved in S. sclerotiorum asexual-to-sexual development (6). To get further insight into the SsNsd1-mediated regulatory mechanism involved in the development of S. sclerotiorum, we used SsNsd1 as bait to search against the S. sclerotiorum cDNA library via a Y2H approach and identified 40 SsNsd1-interacting protein candidates (see Data Set S1 in the supplemental material). Among them, one formaldehyde dehydrogenase (SS1G_10135), SsFdh1, was further confirmed to interact with SsNsd1 in yeast cells (Fig. S1A).

10.1128/mSystems.00397-19.1 FIG S1 Deletion of *Ssfdh1* does not affect hyphal branching or sclerotium number. (A) Y2H showed that SsNsd1 interacted with SsFdh1 in yeast cell. pGADT7-T and pGBKT7-53 were used as positive controls, while pGADT7-T and pGBKT7-lam were used as negative controls. “-” represents empty vector. The yeast transformants were cultured on minimal medium quadruple dropouts (QDO) supplemented with 20 mg ml^−1^ X-α-Gal (5-bromo-4-chloro-3-indolyl-α-d-galactopyranoside). All the photographs were taken 3 days postinoculation (dpi). (B) Model representing the homologous recombination-based gene knockout strategy. The Hyg cassette was used to replace the *Ssfdh1* sequence. (C) PCR test using genomic DNA of WT and KO strains as the template identifying deletion of *Ssfdh1*. DL2000, DL2000 DNA marker. “*Ssfdh1*” indicates the complete 1,979-bp *Ssfdh1* DNA sequence. “*Hyg*” indicates the complete 1,412-bp *hph* gene sequence. “LF+Hy” indicates the 2,022 bp containing the left segment and the partial *hph* sequence. “RR+yg” indicates the 2,018 bp containing the right segment and the partial *hph* sequence. (D) PCR test to identify the complementation of *Ssfdh1* with genomic DNA from WT, KO, and C-22 strains*. “Ssfdh1*” and “*Hyg*” indicates the same sequences as those described for panel C. “*G418*” indicates the partial 672-bp G418 antibiotic gene sequence. (E) Microscopic observation of WT, KO, and C-22 strains. Light microscopy was used to observe the hyphal branching patterns. Scale bar, 10 μm. (F) Sclerotium observation for WT, KO, and C-22 strains on PDA medium. Photographs were taken at 10 DAI. (G) Statistical analysis of WT, KO, and C-22 sclerotium numbers on SPA medium at 10 DAI. Download FIG S1, TIF file, 2.1 MB. Copyright © 2019 Zhu et al. 2019 Zhu et al. This content is distributed under the terms of the Creative Commons Attribution 4.0 International license.

10.1128/mSystems.00397-19.9 DATA SET S1 A list of putative SsNsd1-interacting proteins identified by yeast two-hybrid screens. Download Data Set S1, DOCX file, 0.04 MB. Copyright © 2019 Zhu et al. 2019 Zhu et al. This content is distributed under the terms of the Creative Commons Attribution 4.0 International license.

**FIG 1:**
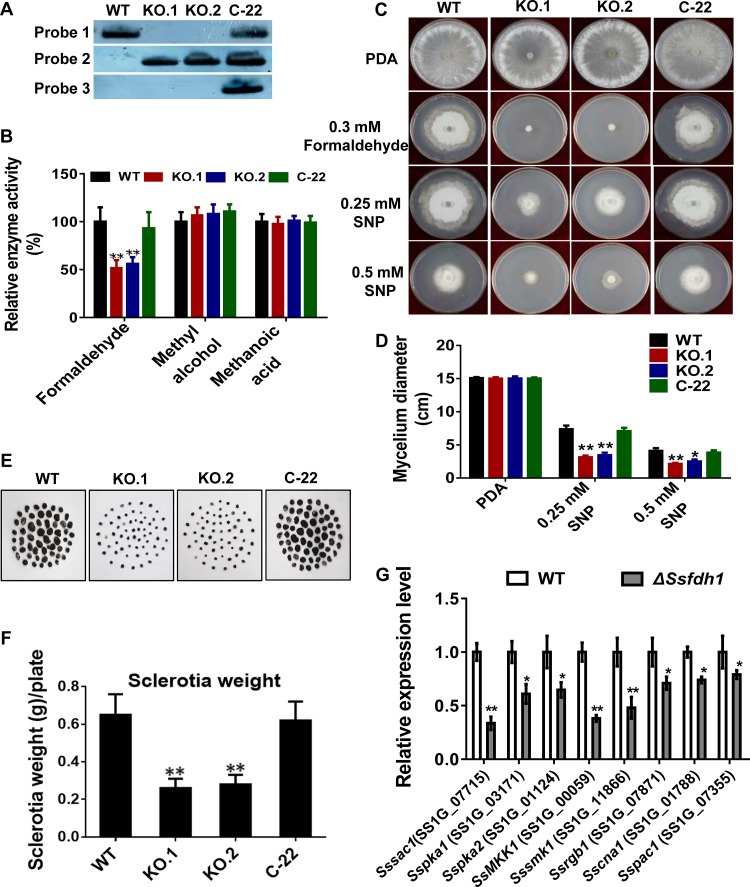
Deletion of *Ssfdh1* associates formaldehyde dehydrogenase activity and sclerotium development. (A) Southern blot assay was performed to validate the presence or absence of selected DNA fragments, such as the *Ssfdh1* genomic DNA region (Probe 1), a partial hygromycin resistance gene (*Hyg*) fragment (Probe 2), and a partial G418 resistance gene fragment (Probe 3). (B) SsFdh1 detoxifies formaldehyde. Relative enzyme activity levels of formaldehyde dehydrogenase were tested with 1.5 ml formaldehyde or methyl alcohol or methanoic acid as a substrate. (C) Deletion of the *Ssfdh1* gene inhibits vegetative growth under conditions of formaldehyde or sodium nitroprusside (SNP) stresses. Mycelium-colonized agar plugs of WT, KO.1, KO.2, and C-22 strains were cultured on PDA medium containing 0.3 mM formaldehyde or 0.25 or 0.5 mM SNP at 28°C for 6 days. (D) Mycelium diameters of colonies of the tested strains were measured, and growth inhibition was evaluated by 6 days after inoculation (DAI). (E) Deletion of *Ssfdh1* affects sclerotium development. Mature sclerotia were collected from a 10-day culture on autoclaved smashed potato agar (SPA) medium at 25°C. (F) Comparison of sclerotium dry weights and numbers among WT, KO, and C-22 strains. Sclerotia were collected from 15-cm-diameter SPA plates for statistics analysis. (G) Expression profiles of sclerotium-associated genes in *Ssfdh1* deletion mutants. Relative expression levels of sclerotium development-associated genes were investigated in hyphal tissue in *Ssfdh1* deletion mutant and WT strains. The constitutively expressed *histone* H3 gene (XM_001589836.1) was used as the reference gene to standardize data. The experiments were performed in triplicate (*, *P < *0.05; ***, *P < *0.01 [Student's *t* test]). Values are means ± standard errors (SE).

*Ssfdh1* knockouts (KOs; KO.1 and KO.2) (Fig. S1B) and genetic complementation strains (C-22), by introduction of an *Ssfdh1* genomic region, were generated in S. sclerotiorum (Fig. 1). We obtained two KOs (Δ*Ssfdh1* KOs; KO.1 and KO.2) and one complementation strain (C22) after molecular confirmation performed with PCR (Fig. S1C and D) and Southern blotting (Fig. 1A). We further observed the hyphal morphology under light microscopy and found that all of the mutants and the control had similar mycelial branching patterns and mycelial convergent states at the early stages (Fig. S1E), suggesting that we could use these strains for further analysis under different conditions.

We assayed enzyme activity with different strains using different substrates. Δ*Ssfdh1* KO strains exhibited significantly impaired FDH activity against formaldehyde compared to the wild type (WT), while introduction of *Ssfdh1* to KO.1 complemented this compromised enzyme activity (Fig. 1B). In this assay, we did not observe any differences among the tested strains under conditions of supplementation of the mycelial crude extract with either methyl alcohol or methanoic acid (Fig. 1B). The FDH activity of SsFdh1 was further characterized by measuring radial hyphal growth in the presence of formaldehyde. In this assay, the growth of KOs was completely inhibited on potato dextrose agar (PDA) medium containing 0.3 mM formaldehyde (Fig. 1C). Again, the introduction of WT *Ssfdh1* into the KO line rescued this phenotype, since the C-22 strain could grow as well as the WT strain (Fig. 1C). The KOs were also evaluated for their ability to resist exogenous NO (nitric oxide) stress. The level of mycelial growth of all of the tested strains was reduced after 6 days on PDA medium containing sodium nitroprusside (SNP), a NO donor. However, the KOs displayed more-severe growth inhibition (Fig. 1C and D). Under these conditions, the growth of strain C-22 was similar to that of the WT strain.

### Deletion of *Ssfdh1* affects sclerotium development.

The sizes and dry weights of mature sclerotia from KOs were significantly lower than those of the WT (Fig. 1E and F), while the numbers of mature sclerotia from KOs were similar to those seen with the WT (Fig. S1F and G). Introduction of *Ssfdh1* complemented the sclerotium development phenotypes (Fig. 1E and F).

We then evaluated the transcript levels of sclerotium development-related genes via reverse transcription-quantitative PCR (qRT-PCR) in the WT and Δ*Ssfdh1* strains. The tested genes included *Sssac1* (19), *Sspka1* and *Sspka2* (20), *SsMKK1* (21), *SsSmk1* (22), *Ssrgb1* (23), *Sscna1* (24), and *Sspac1* (25). During sclerotium development, the expression levels of these genes were significantly reduced in the Δ*Ssfdh1* mutant compared to the WT strain (Fig. 1G).

### *Ssfdh1* is associated with compound appressorium formation and pathogenicity.

**FIG 2:**
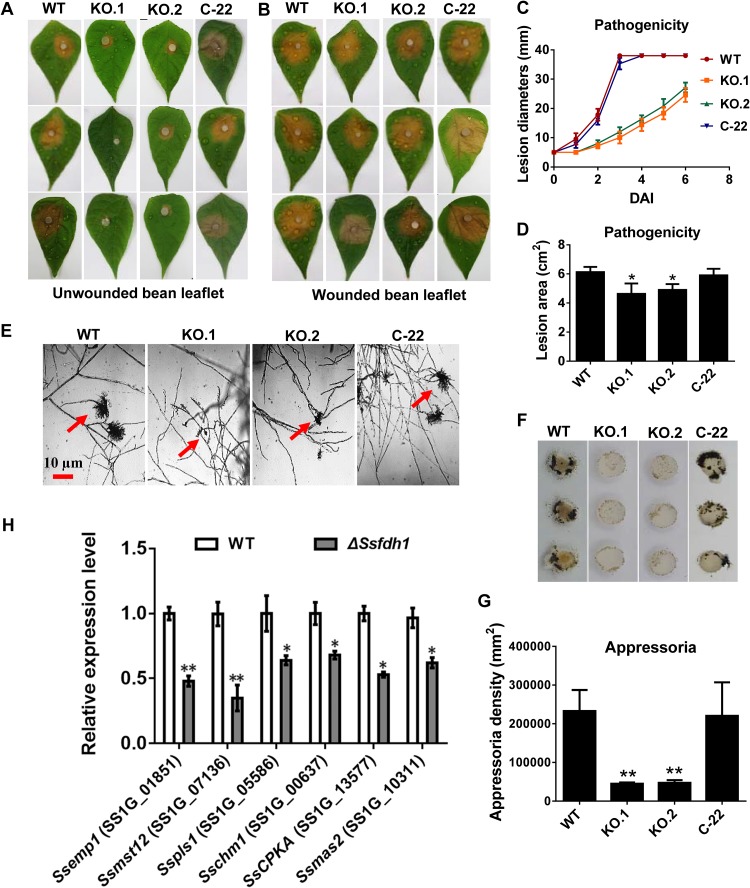
Deletion of *Ssfdh1* attenuates pathogenicity mainly by a deficiency in compound appressoria. (A) WT, KO, and C-22 strains were inoculated on unwounded common bean leaves and photographed 2 days after inoculation (DAI). (B) Pathogenicity phenotype of WT, KO, and C-22 strains on wounded common bean leaves. Pictures were taken by 2 DAI. (C) Lesion diameters were measured on unwounded leaves daily until 6 DAI. (D) Lesion areas were measured on unwounded leaves by 2 DAI. (E) Compound appressoria (red arrows) of WT, KO, and C-22 strains on glass sides were observed by light microscopy at 1 DAI. (F) Compound appressorium development of WT, KO, and C-22 strains on parafilm. Pigmented compound appressoria were observed on parafilm at 1 DAI using 5-mm-diameter mycelial plugs. (G) Quantities of compound appressoria calculated by ImageJ pixel density analysis compared among different strains. (H) Relative expression levels of compound appressorium-associated genes were investigated in hyphal tissue in *Ssfdh1* deletion mutant and WT strains. The constitutively expressed *histone* H3 gene was used as the reference gene to standardize data (*, *P < *0.05; **, *P < *0.01 [Student's *t* test, *n* = 3]).

The virulence of KOs was strongly reduced on healthy bean leaves (Fig. 2A and C) at 2 days after inoculation (DAI). Both KO strains exhibited a decreased ability to infect healthy host leaves compared to the WT and C-22 strains and developed smaller lesion areas (Fig. 2A and C). The development of compound appressoria plays an important role in pathogenicity (5, 6). We found that, at 1 DAI, the KOs had developed fewer pigmented compound appressoria than did the WT and C-22 strains on glass slides or parafilm (Fig. 2E to G). What is more, KO.1 and KO.2 did not produce normal compound appressoria of the same size as those generated by WT and C-22 (Fig. 2E).

We tried to evaluate the transcript levels of infection cushion generation-related genes, such as *Ssemp1*, *Ssmst12*, *Sspls1*, *Sschm1*, *Ssmas2* (26), and *SscpkA* (27), via qRT-PCR in the WT and Δ*Ssfdh1* strains. The mRNA levels of these genes were diminished in the Δ*Ssfdh1* mutant during compound appressorium development (Fig. 2H). We asked whether the impaired pathogenicity on the healthy leaf tissues was caused by impaired infection cushion formation, and we inoculated the wounded common bean leaves with different strains. The inoculation results showed that the KOs successfully infected the wounded leaves in a manner similar to that seen with the WT and C-22 strains (Fig. 2B). However, the lesions produced by the KOs were less extensive (smaller in size) than those produced by the WT and C-22 strains (Fig. 2B and D), indicating that SsFdh1 is also involved in postpenetration pathogenicity.

### *Ssfdh1* is important in osmotic and oxidative stress resistance.

To study how *Ssfdh1* affects penetration-independent pathogenesis-associated stress adaptation, we cultured the WT, KO, and C-22 strains on PDA containing different stress agents. Under osmotic stress conditions, such as 1 M KCl, 1 M NaCl, or 1 M sorbitol, KOs showed inhibited hyphal growth compared to the control strains as determined by analysis of the visible hyphal growth on the plates (Fig. S2A and B). The KOs also showed smaller colony diameters under conditions of SDS treatment, which can destroy cells by dissolving proteins and lipids in the cell membrane (Fig. S2A and B). We also subjected different strains to oxidative stress by adding H_2_O_2_, and we found that when we enhanced the oxidative stress by increasing the concentration of H_2_O_2_ from 5 to 20 mM, the hyphal growth of the WT, the KOs, and C-22 was increasingly inhibited (Fig. S2C and D). However, the KOs were more sensitive to H_2_O_2_ than the control strains under the same treatment conditions (Fig. S2C and D).

10.1128/mSystems.00397-19.2 FIG S2 Deletion of the *Ssfdh1* gene increases sensitivity to osmotic and oxidative stress. (A and B) Morphological observation and colony diameter of WT, KO, and C-22 strains. These strains were inoculated on PDA medium amended with 1 M KCl, 1 M NaCl, 1 M sorbitol, or 0.02% SDS for 3 days. (C and D) Morphological observation (C) and colony diameter (D) of WT, KO, and C-22 strains on PDA medium containing different concentrations of H_2_O_2_. Photographs were taken by 3 DAI. The significant difference is indicated by asterisks (*, *P < *0.05; **, *P < *0.01 [Student’s *t* test, *n* = 3]). Download FIG S2, TIF file, 1.7 MB. Copyright © 2019 Zhu et al. 2019 Zhu et al. This content is distributed under the terms of the Creative Commons Attribution 4.0 International license.

### SsNsd1 directly regulates *Ssfdh1* transcripts.

**FIG 3:**
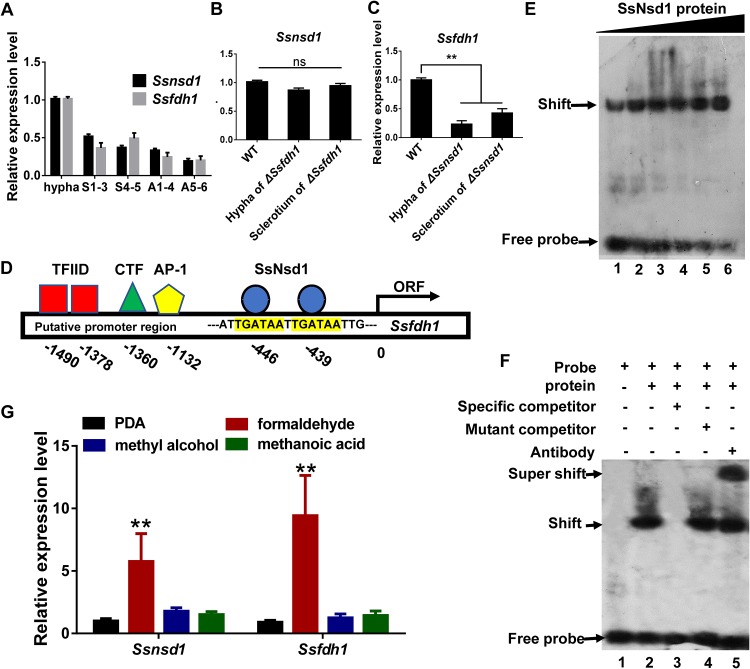
The transcription factor SsNsd1 regulates the expression of *Ssfdh1* by binding the GATA-box region of the putative promoter. (A) The relative expression levels of *Ssfdh1* and *Ssnsd1* in hyphae, sclerotia (S1-3 and S4-5 stages), and apothecia (A1-4 and A5-6 stages). The constitutively expressed *histone* H3 gene (XM_001589836.1) was used as the reference gene to standardize data. (B) *Ssfdh1* expression levels in hypha and sclerotium stages of WT and Δ*Ssnsd1* strains. (C) The *Ssnsd1* expression levels in hypha and sclerotium stages of WT and Δ*Ssfdh1* strains. (D) Schematic diagram showing the predicted binding elements in the promoter region upstream of *Ssfdh1* coding sequence. Predicted binding elements include TFIID (TATA transcription factor), CTF (CCAAT transcription factor), AP-1 (bZIP transcription factor), and SsNsd1 (GATA transcription factor). The transcription start sites are indicated as “0.” ORF, open reading frame. (E) Characterization of SsNsd1 GATA-binding interaction by electrophoretic mobility shift assays (EMSA). A quantity of SsNsd1 protein (500 ng, 600 ng, 700 ng, 800 ng, 900 ng, or 1,000 ng) was incubated with a 30-bp *Ssfdh1* promoter probe. With the increase in the SsNsd1 amount, more DNA shift was observed with less free probe accumulation at the bottom. (F) Further confirmation of SsNsd1 GATA-box binding. The labeled DNA probe was preincubated with 500 ng SsNsd1, and then a 100-fold excess of unlabeled special competitor (unlabeled DNA probe), a 100-fold excess of unlabeled mutant competitor (unlabeled mutant DNA probe), or 1 μg anti-GATA TF commercial antibody was added. The presence or absence of the reaction content is indicated with a minus sign or a plus sign, respectively. (G) *Ssnsd1* and *Ssfdh1* expression levels of the WT strain in the mycelial stage of growth was tested by qRT-PCR. The WT strain was cultured on the PDA medium with formaldehyde, methyl alcohol, or methanoic acid. The *histone* H3 gene was used as the reference gene to standardize the data. Statistically significant differences are indicated by asterisks (**, *P < *0.01 [Student's *t* test, *n* = 3]).

We profiled the expression levels of *Ssnsd1* and *Ssfdh1* during different developmental stages and found that the expression levels of *Ssnsd1* and *Ssfdh1* were spatially and temporally synchronized (Fig. 3A). Both *Ssnsd1* and *Ssfdh1* showed higher transcription accumulation levels in the hyphal stage than in the sclerotium (S1 to S5) and apothecium (A1 to A6) stages (Fig. 3A). To explore the genetic relationship between *Ssnsd1* and *Ssfdh1*, we determined the levels of expression of both genes in different genetic backgrounds by qRT-PCR. The qRT-PCR results showed that the expression levels of *Ssnsd1* were similar in the WT and *ΔSsfdh1* strains (Fig. 3B), whereas the transcription level of *Ssfdh1* in the *ΔSsnsd1* strain was drastically reduced relative to the WT (Fig. 3C), suggesting that SsNsd1 regulates *Ssfdh1* transcripts. SsNsd1 contains a zinc finger (ZnF) GATA binding domain at the region spanning amino acids (aa) 320 to 385 of its C terminus, which may recognize and bind a core (A/T)GATA(A/G) consensus sequence (11). We then scanned a 2-kb *Ssfdh1* promoter region and identified two core GATA sequences located at nucleotides −446 and −439 (Fig. 3D; see also Data Set S2).

10.1128/mSystems.00397-19.10 DATA SET S2 Ssfdh1 coding sequence and its putative promoter region (coding sequences are colored green, and the promoter region is colored blue). Download Data Set S2, DOCX file, 0.03 MB. Copyright © 2019 Zhu et al. 2019 Zhu et al. This content is distributed under the terms of the Creative Commons Attribution 4.0 International license.

Next, we asked whether SsNsd1, as a GATA TF, could directly bind to the GATA sequences in the *Ssfdh1* promoter region. We performed an *in vitro* electrophoretic mobility shift assay (EMSA) with a probe containing two predicted GATA consensus sequences as indicated in Fig. 3D. SsNsd1 increasingly bound to the DNA probe along with increasing recruitment of SsNsd1 protein (Fig. 3E). In the full EMSA, SsNsd1 efficiently bound to the GATA-box DNA probe (Fig. 3F, lane 2). The specificity of the SsNsd1-DNA complex was confirmed by adding an excess of unlabeled DNA (Fig. 3F, lane 3). As expected, a shift was detected when the unlabeled DNA was mutated (Fig. 3F, lane 4). We used commercial anti-GATA TF antibody to visualize the super shift of the protein-DNA complex (Fig. 3F, lane 5), and the results clearly showed a supershifted band. These data suggest that SsNsd1 can directly bind to the GATA sequence in the *Ssfdh1* promoter. qRT-PCR evaluation of *Ssfdh1* and *Ssnsd1* transcripts in WT hypha treated with formaldehyde, methyl alcohol, and methanoic acid showed that both *Ssnsd1* and *Ssfdh1* had significantly higher mRNA levels after formaldehyde treatment (Fig. 3G). Together, these data suggest that SsNsd1 has the potential to directly regulate the *Ssfdh1* gene by recognizing its GATA-box.

### Overexpression of *Ssfdh1* in the Δ*Ssnsd1* strain partially restored the sclerotia and compound appressorium deficiency.

**FIG 4:**
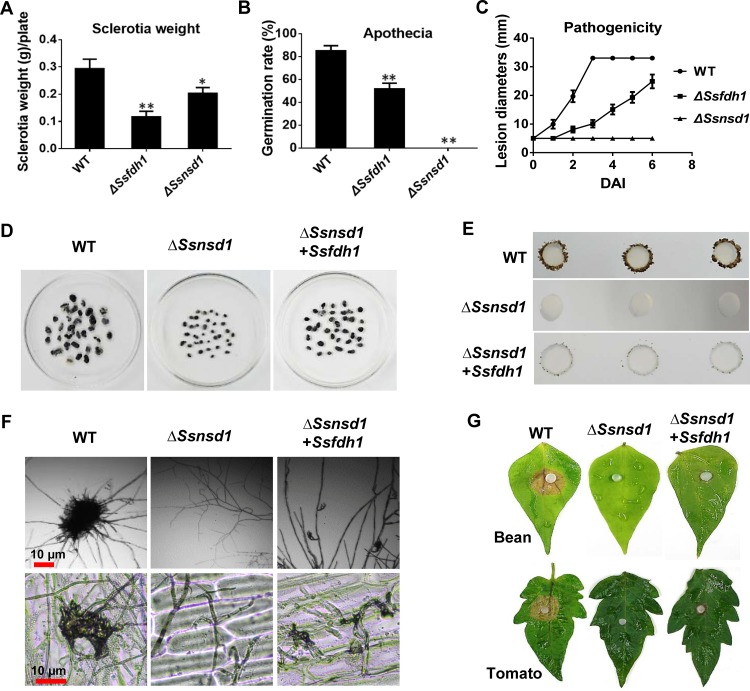
Overexpression of *Ssfdh1* in the Δ*Ssnsd1* mutant does not restore the deletion phenotype of SsNsd1. (A, B, and C) Phenotype comparisons of *Ssfdh1* and *Ssnsd1* deletion mutants. The phenotypes of WT, Δ*Ssfdh1*, and Δ*Ssnsd1* strains were analyzed at different development stages, and sclerotium weights (A), apothecial germination rates (B), and pathogenicity (C) on unwounded bean leaflets were measured. Sclerotia were collected from 9-cm-diameter PDA plates for statistics analysis. The same experiments were repeated three times. Significant differences are indicated by asterisks (*, *P < *0.05; ***, *P < *0.01 [Student's *t* test]). (D, E, F, and G) Phenotype analyses of *Ssfdh1* overexpression in the *Ssnsd1* deletion mutant background (Δ*Ssnsd1* + *Ssfdh1*). (D) Comparison of sclerotia dry weights of WT, Δ*Ssnsd1*, and Δ*Ssnsd1* + *Ssfdh1* strains. Sclerotia were collected from 15-cm-diameter plates for statistics analysis. (E) Compound appressorium development of WT, Δ*Ssnsd1*, and Δ*Ssnsd1* + *Ssfdh1* strains on parafilm. Pigmented compound appressoria were observed at 3 DAI using 5-mm-diameter mycelial plugs. (F) Compound appressoria of WT, Δ*Ssnsd1*, and Δ*Ssnsd1* + *Ssfdh1* strains were observed on glass sides or onion epidermal strips cell by light microscopy. (G) Pathogenicity phenotype of WT, Δ*Ssnsd1*, and Δ*Ssnsd1* + *Ssfdh1* strains on unwounded bean or tomato leaves. Pictures were taken by 2 DAI when the lesions were observed under the agar plug.

We sought additional evidence for SsNsd1 regulation of *Ssfdh1* by comparing development-associated phenotypes of WT, Δ*Ssfdh1*, and Δ*Ssnsd1* strains. The *Ssnsd1* and *Ssfdh1* deletion mutants shared similar vegetative developmental phenotypes (Fig. 4A to C). Phenotypically, both the Δ*Ssnsd1* and *ΔSsfdh1* strains exhibited defective development with respect to the size and dry weight of sclerotia (Fig. 4A; see also Fig. 1E). The WT had a high (85%) carpogenic germination rate, but only about half of the sclerotia from the *ΔSsfdh1* mutant formed apothecia, while the *ΔSsnsd1* mutant lacked the ability to produce apothecia (Fig. 4B) (6). With regard to pathogenicity, the WT strain had fully colonized detached healthy leaves by 3 DAI, while colonization was delayed by 3 to 4 days in the *ΔSsfdh1* mutant and the *ΔSsnsd1* mutant was nonpathogenic on unwounded host tissue (Fig. 4C).

We then expressed the SsFdh1 protein under the control of a strong *OliC* promoter in the Δ*Ssnsd1* background and found that overexpression of *Ssfdh1* did not provide full rescue but did restore some of the deficiencies in production of sclerotia and compound appressoria (Fig. 4D and E). The Δ*Ssnsd1* mutant was unable to generate compound appressoria (6), while overexpression of *Ssfdh1* partially restored the loss of compound appressoria (Fig. 4F). Consequently, overexpressing *Ssfdh1* in the Δ*Ssnsd1* background allowed host penetration but symptom development was delayed (Fig. 4G) .

### SsNsd1 interacted with SsFdh1 in a disulfide bond-dependent manner.

**FIG 5:**
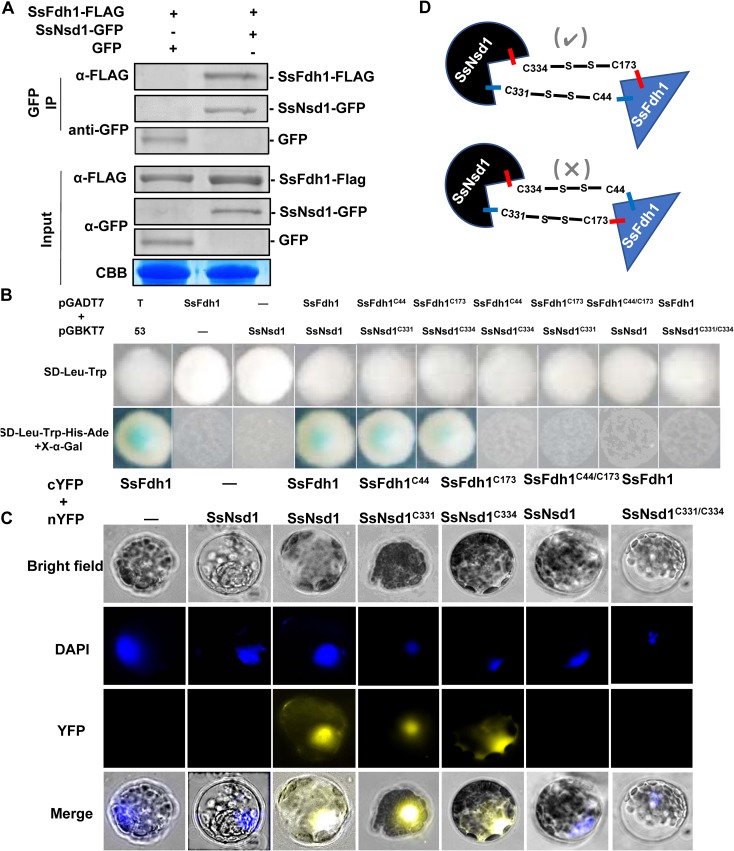
SsFdh1 interacts with SsNsd1 through Cys residues in the zinc finger motif. (A) SsFdh1 interacts with SsNsd1 in coimmunoprecipitation (Co-IP) assay. SsNsd1-GFP/empty vector (control) and SsFdh1-FLAG were expressed in N. benthamiana leaves under conditions of *Agrobacterium*-mediated transient expression. Anti-GFP IP was performed, and the interaction was visualized by Western blotting. CBB, Coomassie brilliant blue. (B) SsNsd1 associates with SsFdh1 through conserved Cys residues. The Y2H assay was performed with different versions of SsNsd1 (WT, C331, and C334) and SsFdh1 (WT, C44, and C173). pGADT7-T and pGBKT7-53 were used as positive controls. Minus signs represent empty vector. (C) Confirmation of the interaction between SsNsd1 and SsFdh1 by bimolecular fluorescence complementation assay (BiFC) in *Arabidopsis* protoplast. Different versions of SsNsd1 and SsFdh1 (the same as those used as described for panel B) were cotransfected into *Arabidopsis* protoplast. Images were captured under a fluorescence microscope at 14 h after *Arabidopsis* protoplast transformation. The nucleus was visualized using DAPI (4′,6-diamidino-2-phenylindole) fluorescent dye. cYFP, C-terminal region of YFP; nYFP, N-terminal region of YFP. (D) Schematic view of two assumed SsNsd1-SsFdh1 interaction modes mediated by disulfide bond between the Cys residues in the zinc finger motifs. The experimentally proven interaction model is indicated by a check mark as determined on the basis of results from the experiments described for panels B and C.

To further confirm the interaction between SsFdh1 and SsNsd1, we transiently coexpressed SsFdh1-3xFLAG and SsNsd1-green fluorescent protein (SsNsd1-GFP) in Nicotiana benthamiana leaves and immunoprecipitated the SsNsd1-GFP with GFP-IP and co-IP results showed that SsFdh1 associates with SsNsd1 (Fig. 5A).

To study how SsNsd1 interacts with SsFdh1 in S. sclerotiorum, the three-dimensional (3D) structures of SsFdh1 and SsNsd1 were modeled. SsFdh1 (aa 8 to 374) was predicted to belong to the Zn-dependent dehydrogenase family due to BLAST homology results in which a zinc ion was found in the predicted structure (Fig. S3A) (28). The active sites of ADH were predicted with two divalent zinc ion-cysteine sites (Cys 44 and Cys 173) that aid catalytic activities and contribute to substrate binding (29) and that are strictly conserved in the SsFdh1 protein (Fig. S3A). The modeled SsNsd1 showed a ZnF module possessing four cysteine residues (Cys 331, Cys 334, Cys 353, and Cys 356) coordinated to a single zinc ion (Fig. S3B) which can stabilize protein structure and function in DNA binding or in protein-protein interactions (30). The ZnF domain is involved in interactions with other ZnF proteins (31). Thus, we assumed that the cysteine residues in this region were crucial for their functions and interaction. Since cysteine-rich protein can form disulfide bonds, we hypothesized SsNsd1 might interact with SsFdh1 through disulfide bond formation. To test this hypothesis, we mutated the cysteine to an alanine residue (C to A) in SsNsd1 and SsFdh1 (Fig. S4A). As the SsNsd^C353/C356^ mutations still maintained the interaction with SsFdh1 (Fig. S4B), Cys residues C353 and C356 of SsNsd1 did not contribute to this interaction. We examined the possibility that C331 and C334 were involved in the interaction (Fig. 5B and C). In yeast, SsFdh1^C44^ interacted with SsNsd1^C331^, but not with SsNsd1^C334^, and SsFdh1^C173^ interacted with SsNsd1^C334^, but not with SsNsd1^C331^ (Fig. 5B). The mutant with the SsFdh1^C44/C173^ double mutation did not maintain the interaction with SsNsd1, and the SsNsd1^C331/C334^ double mutant was not able to interact with SsFdh1 (Fig. 5B). These results were further confirmed in Arabidopsis thaliana protoplast with a bimolecular fluorescence complementation (BiFC) assay (Fig. 5C). These results confirmed the SsFdh1-SsNsd1 interaction and indicated that the correct interaction was mediated by disulfide bonds (SsFdh1^C44-S-S-C331^SsNsd1 and SsFdh1^C173-S-S-C331^SsNsd1) as shown in Fig. 5D.

10.1128/mSystems.00397-19.3 FIG S3 Zinc finger motif prediction using three-dimensional model of SsFdh1 and SsNsd1. (A) Molecular three-dimensional structure of SsFdh1 protein (aa 8 to 374). α-Helices are colored in cyan, and β-strands are colored in magenta. A zinc ion was predicted to localize in the zinc finger motif. The conserved Cys 44 and Cys 173 residues were predicted to be coordinated with a zinc ion in the active site. (B) Three-dimensional structure of SsNsd1 protein (aa 320 to 385). The conserved Cys 331, Cys 334, Cys 353, and Cys 356 residues were coordinated with a zinc ion in the zinc finger motif. Download FIG S3, TIF file, 1.5 MB. Copyright © 2019 Zhu et al. 2019 Zhu et al. This content is distributed under the terms of the Creative Commons Attribution 4.0 International license.

10.1128/mSystems.00397-19.4 FIG S4 The point mutation strategy for Cys residues and SsFdh1 and SsNsd1^C353/C356^ Y2H results. (A) Point mutation strategy. Cys^331^, Cys^334^, Cys^353^, or Cys^356^ of SsNsd1 was replaced with residue Ala by changing nucleotide sequences TGT (Cys^331^/Cys^356^) and TGC (Cys^334^/Cys^353)^ to GCT (Ala) and GCC (Ala), respectively. Cys^44^ and Cys^173^ of SsFdh1 were replaced with Ala by mutating nucleotide sequence TGT (Cys^44^ and Cys^173^) to GCT (Ala). PCR was used to generate the point mutation constructs. (B) Y2H showed that SsNsd1^C353/C356^ did not associate with SsFdh1 in yeast cells. pGADT7-SsFdh1 and pGBKT7-SsNsd1 were used as positive controls. “-” represents empty vector. The yeast transformants were cultured on minimal medium quadruple dropouts (QDO) supplemented with 20 mg ml^−1^ X-α-Gal. All the photographs were taken 3 days postinoculation (dpi). Download FIG S4, TIF file, 1.9 MB. Copyright © 2019 Zhu et al. 2019 Zhu et al. This content is distributed under the terms of the Creative Commons Attribution 4.0 International license.

### SsNsd1 enriched SsFdh1 in the nucleus.

**FIG 6:**
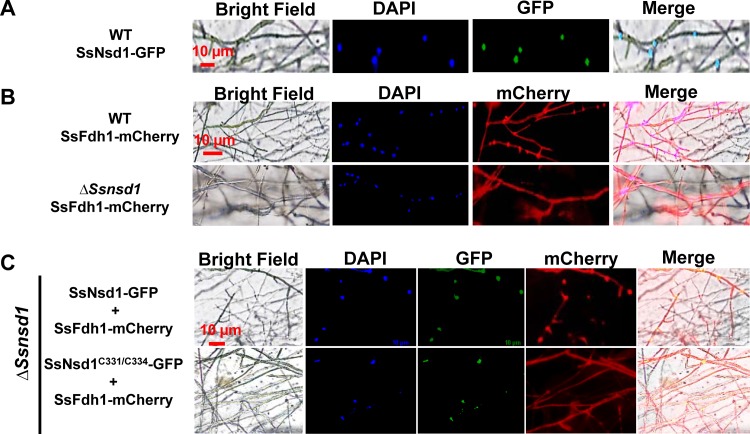
The SsNsd1-SsFdh1 interaction promotes the enrichment of SsFdh1 in the nucleus. (A) The SsNsd1-GFP fusion protein localizes in nucleus in growing hyphae of S. sclerotiorum. (B) Expression of SsFdh1-mCherry fusion protein in the strain WT or Δ*Ssnsd1* background strains. (C) Coexpression of SsFdh1-mCherry and SsNsd1-GFP (or SsNsd1^C331/C334^-GFP) fusion protein in Δ*Ssnsd1* background strains. The SsNsd1-GFP fusion protein or SsFdh1-mCherry fusion protein was expressed under the control of the *oliC* promoter in S. sclerotiorum strains. The nucleus was stained with DAPI. Scale bars are indicated in the images.

Our BiFC results showed that SsFdh1 interacted with SsNsd1 in the nucleus (Fig. 5C). However, results obtained using a protein subcellular localization prediction tool (PSORT) indicated that SsNsd1 is a nucleus-localized protein and SsFdh1 was predicted to be cytoplasmic. We hypothesized that the interaction between SsNsd1 and SsFdh1 might change the native subcellular localization of SsFdh1. To test this hypothesis, a localization assay was performed in S. sclerotiorum. First, SsNsd1-GFP was expressed in hyphae of the WT strain and SsNsd1 was clearly observed to localize in the nucleus (Fig. 6A). We found that SsFdh1 localized in the nucleus and cytoplasm when SsFdh1-mCherry was expressed in WT hyphae but that SsFdh1 was mostly localized in the cytoplasm when expressed in the Δ*Ssnsd1* mutant (Fig. 6B)* .* This result illustrated that SsNsd1 recruits SsFdh1 to the nucleus. SsNsd1-GFP and SsFdh1-mCherry were then coexpressed in the Δ*Ssnsd1* mutant (Fig. 6C)* .* SsNsd1 was localized in the nucleus, and SsFdh1 exhibited both nuclear and cytoplasmic localization (Fig. 6C). When SsNsd1^C331/C334^-GFP and SsFdh1-mCherry were coexpressed in the Δ*Ssnsd1* mutant, Nsd1^C331/C334^ was still localized in the nucleus, but SsFdh1 localized to the cytoplasm. This appears to have been due to its inability to interact with SsFdh1 (Fig. 6C).

To test whether stress conditions can change the interaction between SsFdh1 and SsNsd1, coexpression strains were exposed to formaldehyde or H_2_O_2_ (Fig. S5)_._ SsFdh1 was still found in the nucleus of compound appressoria when coexpressed with SsNsd1 under conditions of formaldehyde or H_2_O_2_ stress (Fig. S5A). However, this nuclear localization was lost when SsNsd1^C331/C334^ -GFP and SsFdh1-mCherry were coexpressed in the Δ*Ssnsd1* mutant under the same stress conditions. The same results were seen in the hyphae of the Δ*Ssnsd1* mutant (Fig. S5B).

10.1128/mSystems.00397-19.5 FIG S5 Nuclear translocation of SsFdh1 mediated by the SsNsd1-SsFdh1 interaction is stable under conditions of stresses in vegetative hyphae (A) and compound appressoria (B) during penetration of onion epidermal cell (C) and postinfection (D). Coexpression of SsFdh1-mCherry and SsNsd1-GFP (or SsNsd1^C331/C334^-GFP) fusion protein in the Δ*Ssnsd1* background strains was analyzed by adding 0.1 mM formaldehyde or 10 mM H_2_O_2_. Bright-field and GFP images were merged (merged1 images), and merged1 and mCherry were merged (merged2 images). The scale bars are indicated in the images. Download FIG S5, TIF file, 2.2 MB. Copyright © 2019 Zhu et al. 2019 Zhu et al. This content is distributed under the terms of the Creative Commons Attribution 4.0 International license.

To study the interaction of SsFdh1 and SsNsd1 during the infection process, the coexpression strains were inoculated on onion epidermal cells. SsFdh1 could be observed in the nucleus of compound appressoria induced on onion epidermal cells. This nucleus localization was lost when SsNsd1^C331/C334^-GFP and SsFdh1-mCherry were coexpressed in the Δ*Ssnsd1* mutant (Fig. S5C). A similar result was observed in the hyphae of the Δ*Ssnsd1* mutant (Fig. S5D).

### SsFdh1 interacts with SsNsd1 and prevents SsNsd1 binding to DNA.

**FIG 7:**
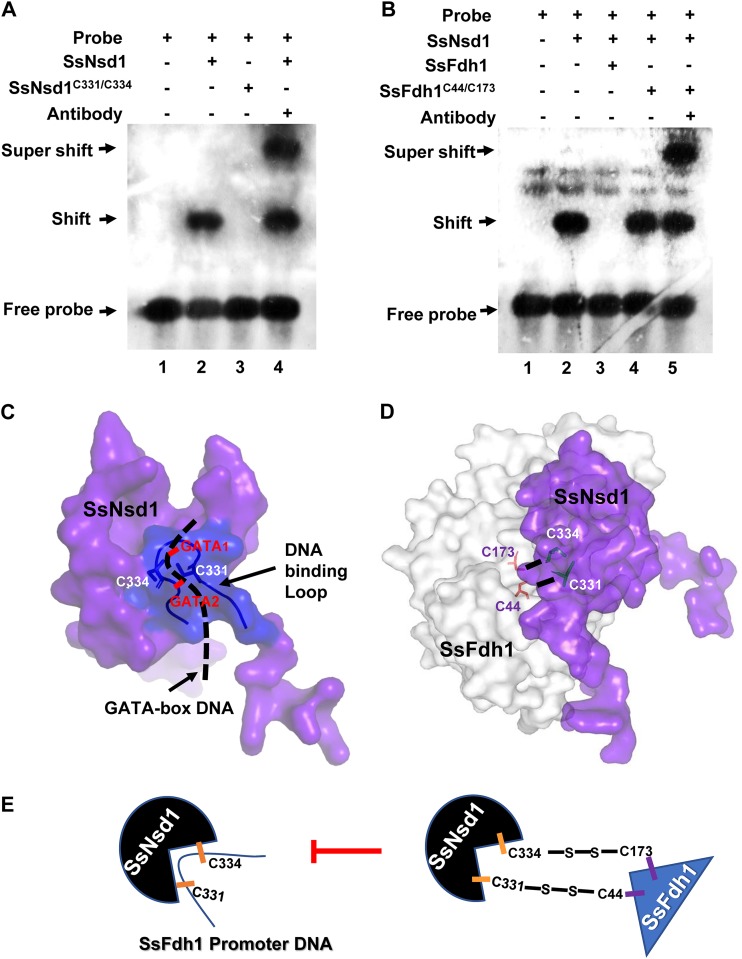
The SsNsd1-SsFdh1 interaction prevents SsNsd1 binding to GATA-box. (A) Cys^331^ and Cys^334^ of SsNsd1 are the key GATA binding sites confirmed by EMSA. The labeled GATA-box DNA probe was incubated with 500 ng SsNsd1, 500 ng Cys^331^/Cys^334^ mutation protein SsNsd1^C331/C334^, or 1 μg anti-GATA TF commercial antibody. (B) SsFdh1 inhibits SsNsd1 GATA-box binding based on the EMSA results. The DNA-labeled probe was preincubated with 500 ng SsNsd1, and then 10 μg SsFdh1, 10 μg Cys^44^/Cys^173^ mutation protein SsFdh1^C44/C173^, or 1 μg antibody was added. The presence or absence of the reaction component is indicated with a plus sign or a minus sign, respectively. (C) SsNsd1 GATA-box binding models and the key Cys^331^ and Cys^334^ residues in the zinc finger motif according to predicted protein structure and EMSA results. The ribbon represents the DNA binding loop of the fungal GATA factor. The black dashed line represents the GATA-box DNA. (D) Schematic representation of SsNsd1-SsFdh1 interaction models and the key Cys^331^, Cys^334^, Cys^44^, and Cys^173^ residues based on the predicted 3D structure and on results shown in Fig. 5. (E) A representative model of how the interaction of SsNsd1 and SsFdh1 could inhibit SsNsd1 GATA-box binding.

We performed *in vitro* EMSA with a GATA-box DNA probe such as we used in the experiments represented by Fig. 3, with SsNsd1 WT protein, and with SsNsd1 protein with point mutations. SsNsd1^C331/C334^ lost the capacity to bind DNA (Fig. 7A). ZnFs are generally regarded as DNA-binding motifs; however, some reports have implicated ZnFs in the mediation of protein-protein interactions (11) and ZnF domains interacting with a protein might lose the ability to bind DNA (31). To test whether the SsFdh1-SsNsd1 association interferes with SsNsd1-DNA binding, GATA probe, SsNsd1 protein, SsFdh1, and SsFdh1^C44/C173^ were tested with the EMSA. Panel B of Fig. 7 shows that SsNsd1 efficiently bound to the GATA probe as described for Fig. 3. In contrast, surprisingly, the coexistence of SsFdh1 and SsNsd1 completely eliminated the DNA affinity of SsNsd1 and no shift band was observed (Fig. 7B, lane 3). Disruption of the SsFdh1-SsNsd1 interaction by point mutations (SsFdh1^C44/C173^) allowed DNA binding of SsNsd1 (Fig. 7B, lane 4). We also observed clearly supershifted bands when we used anti-GATA TF commercial antibody (Fig. 7B, lane 5). These data suggest that the SsNsd1-SsFdh1 association interferes with SsNsd1-DNA binding.

We then predicted a 3D structure using the SsNsd1-DNA binding model and a simulated pattern diagram of SsNsd1 interacting with SsFdh1 by hypothetically combining their individual protein models (Fig. 7C and D). The results showed that SsFdh1 binding to SsNsd1 protein blocks efficient recognition of or binding to GATA-box DNA by GATA factor SsNsd1 (Fig. 7E).

### The interaction core Cys residues are essential for function of SsFdh1 and SsNsd1.

**FIG 8:**
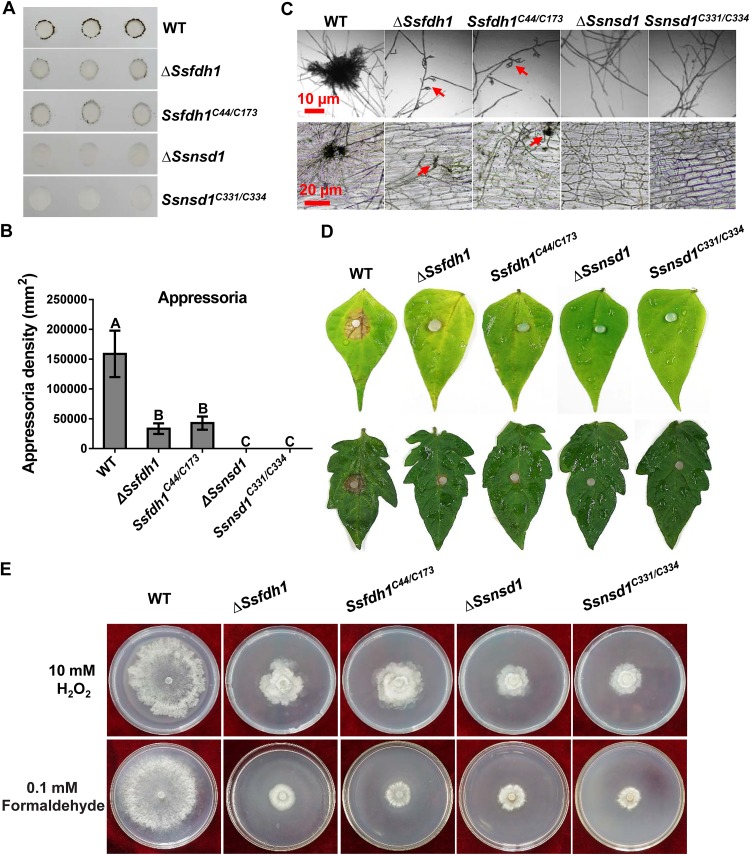
Cys^331^/Cys^334^ and Cys^44^/Cys^173^ in the zinc finger motifs are important for SsNsd1 and SsFdh1 function. (A) Compound appressorium assay. Compound appressorium development of WT, Δ*Ssnsd1*, *Ssnsd1^C331/C334^*, Δ*Ssfdh1* and *Ssfdh1^C44/C173^* strains on parafilm at 3 DAI using 5-mm-diameter mycelial plugs was assayed. (B) Quantification of compound appressoria. The compound appressorium numbers were calculated by ImageJ pixel density analysis. Three independent experiments were performed. Data are represented as mean values ± SE. Different letters indicate statistical significance (*P = *0.05). (C) Compound appressoria of WT, Δ*Ssnsd1*, *Ssnsd1^C331/C334^*, Δ*Ssfdh1*, and *Ssfdh1^C44/C173^* strains were observed by light microscopy at 1 DAI. Compound appressoria are indicated by red arrows. Scale bars are shown in the images. (D) Pathogenicity phenotype of these strains on unwounded bean or tomato leaves. Pictures were taken by 2 DAI while the lesions were first observed under the colonized agar plug of the Δ*Ssfdh1* strain. (E) Growth observations of WT, Δ*Ssnsd1*, *Ssnsd1^C331/C334^*, Δ*Ssfdh1*, and *Ssfdh1^C44/C173^* strains on PDA medium containing 10 mM H_2_O_2_ or 0.1 mM formaldehyde. Photographs were taken by 3 DAI.

The four cysteine residues play crucial roles in SsNsd1-SsFdh1 interactions. To study the biological relevance of these cysteine residues, knockout mutants and corresponding strains complemented with double point mutations were phenotypically analyzed during compound appressorium development and stress resistance (Fig. 8). In the context of compound appressorium development and pathogenicity, the KO strains complemented with Cys mutations could not rescue the development and pathogenicity deficiency phenotypes, since those complementations exhibited effects on compound appressorium development and pathogenicity similar to those seen with the KOs (Fig. 8A to D). In addition, point mutations of SsFdh1 or SsNsd1 had similar levels of sensitivity to H_2_O_2_ and formaldehyde stress (Fig. 8E). These results demonstrate that core Cys residues play a role in biological functions that is as important as their role in maintaining the interactions of SsFdh1 and SsNsd1.

## DISCUSSION

We previously characterized the functions of SsNsd1 in S. sclerotiorum (6) in the context of sclerotium development and pathogenicity. In this study, we identified SsFdh1 as an SsNsd1-interacting protein using the Y2H method and this interaction was further confirmed by different methods (Fig. 5; see also Fig. S1A). SsFdh1 is classified as a glutathione-dependent FDH with common activity involving detoxification of endogenous and exogenous formaldehyde (32). As expected, the Δ*Ssfdh1* mutants showed lethal phenotypes in the presence of formaldehyde stress (Fig. 1). FDHs also have S-nitrosoglutathione (GSNO) reductase activity (17), controlling GSNO and S-nitrosothiol (SNO) levels in addition to detoxifying formaldehyde in cells (33). In yeast, *fdh* mutant cells show increased susceptibility to nitrosative challenges, indicating that GD-FDHs provide protection against nitrosative stress (15). In line with this, we also observed that *Ssfdh1* mutants displayed greater sensitivity to exogenous SNP stress than the controls, as did their counterpart in filamentous fungus M. oryzae (Fig. 1) (14). Endogenous NO in fungi can also be involved in conidiation germination (34) and in formation of infection structures (35). Thus, loss of *Ssfdh1* might affect growth and development by affecting *Ssfdh1*-mediated NO metabolism during these biological processes.

Sclerotium development involves several distinct stages (36) and is tightly regulated by many intrinsic genetic factors. Losing the capacity to produce normal sclerotia in S. sclerotiorum could disrupt the disease cycle and the ability to cause disease (6, 23, 25). We found that the Δ*Ssfdh1* mutant lost its capability to produce normal sclerotia and compound appressoria and showed severely impaired pathogenicity compared to the control strains (Fig. 2). Similarly, after deletion of its homologous protein, MoSFA1, M. oryzae could still form appressoria, whereas the turgor pressure and virulence of appressoria were severely reduced (14). The Δ*Ssfdh1* mutant produced lesions that were less extensive than those produced by the WT and C-22 strains on wounded host, indicating a penetration-independent virulence strategy (Fig. 2B). To cope with pathogen infection, production of reactive oxygen species (ROS) and of reactive nitrogen species (RNS) is one of a variety of mechanisms that plants have evolved to combat pathogen attack (37). Thus, plant pathogens encounter stresses imposed by hosts upon penetration into host cells, and the resistance or adaptation of pathogens to these stresses has been widely regarded as contributing to pathogenesis (38). We observed attenuated virulence in host infection with *Ssfdh1* KO mutants, and those KOs showed significantly decreased resistance to stress agents, such as salts, oxidants, and SDS (Fig. S2). This indicated that SsFdh1 might play an important role in infection-associated stresses beyond formation of compound appressoria.

Nitric oxide (NO) is a short-lived, endogenously produced radical that acts as a signaling molecule (39), and GD-FDHs with GSNO reductase activity, critical for nitrosative stress, are evolutionarily present in yeast, humans, and plants (17). For example, elaborate enzymatic defenses against the nitrosative stress mounted by the host are used to promote fungal virulence of Cryptococcus neoformans (18). *MoSFA1*-mediated NO metabolism functions in redox homeostasis and protein S-nitrosylation in response to development and host infection of M. oryzae (14). In this study, the Δ*Ssfdh1* mutant showed sensitivity to SNP stress, confirming its GSNO reductase activity. Given the positive roles of FDHs in protecting pathogens against infection-associated stresses, SsFdh1 could help overcome host defense and increase S. sclerotiorum pathogenicity. This result supports the attenuated virulence of the mutants observed on the wounded host (Fig. 2B).

The GATA-type ZnF factor NsdD regulates the early stages of the sexual reproduction pathway in A. nidulans (40, 41). An important and complex NsdD signaling pathway involving brlA, rosA, nosA, nsdB, and NsdD in A. nidulans has been shown to regulate sexual and asexual development, pathogenicity, and physiology (41, 42). In S. sclerotiorum, deletion of *Ssnsd1* reduced the expression of *Ssfdh1* (Fig. 3), indicating that SsFdh1 might function as a downstream target regulated by SsNsd1. In support of this, SsNsd1 could directly bind to the GATA-box DNA in EMSA (Fig. 3). However, binding sites of other TFs, such as TFIID, CTF, and AP-1, were also found in the *Ssfdh1* promoter region, indicating that other TFs or other GATA factors might be involved in the regulation of *Ssfdh1* (Fig. 3). This accounted for the detectable expression of *Ssfdh1* in the Δ*Ssnsd1* mutant. Similarly, NsdD repressed conidiation by binding to the GATA-box upstream of *brlA*, but loss of *flbC* or *flbD* in the absence of nsdD resulted in delayed activation of *brlA*, suggesting positive roles of other activators in conidiation development of A. nidulans (42). Deletion of *Ssfdh1* produced only partial developmental phenotypes compared to the *Ssnsd1* mutants, while overexpression of *Ssfdh1* did not restore the full function of the *Ssnsd1* mutant, indicating that *Ssfdh1* was only one of the downstream genes that SsNsd1 could regulate during sclerotium development, compound appressorium formation, and pathogenicity. In A. flavus, NsdD is essential for transcriptional control of a series of genes that are vital for the morphological development of sclerotia and the production of conidia (43). In this regard, SsNsd1could also regulate several downstream targets during compound appressorium formation (12).

ZnFs can act as protein-protein recognition platforms in addition to their familiar role as DNA recognition motifs (30). In vertebrates, the GATA-1 factors contain two ZnF domains, termed the N-finger and the C-finger domains. Generally, the C-finger domain is involved in sequence-specific DNA binding, and the N-finger is involved in the mediation of protein-protein interactions with other ZnF proteins (44). In fungi, the GATA factors contain only one DNA-binding domain (11). We confirmed that SsNsd1 could bind GATA-box DNA *in vitro* and that SsNsd1 physically associates with SsFdh1 in a disulfide bond-dependent manner. Mutagenesis analysis reveals the importance of Cys residues in the ZnF domain in both proteins. When mutated (C331/C334), SsNsd1 could neither bind to the GATA-box sequence from Ssfdh1 promoter region nor associate with SsFdh1, clarifying the mechanism by which ZnF proteins might interact with other ZnF proteins. It is likely that fungal GATA proteins utilize the same ZnF domain for both DNA and other ZnF protein binding. This dual role appears to be important for regulation as the SsNsd1-SsFdh1 interaction occurs in the nucleus and prevents SsNsd1 GATA-box binding. SsNsd1 recruits SsFdh1 to the nucleus by physical interactions that might prevent the binding of SsNsd1 to its target DNA regions, while SsNsd1 regulates Ssfdh1 transcriptionally, suggesting the presence of a regulatory loop by which the SsNsd1-SsFdh1 interaction and nuclear localization can interfere with the SsNsd1 factor to activate or repress downstream genes. However, we observed consistent phenotypes in *Ssnsd1* and *Ssfdh1* deletion mutants, indicating that SsNsd1 functions partially through SsFdh1 and that the expression of *Ssfdh1* is regulated by other unidentified factors. Similarly, two ZnFs of EVI1 (ecotropic viral integration site 1) protein have been demonstrated to interact with the ZnF of GATA1 and compete with GATA1 for DNA-binding sites, resulting in repression of gene activation by GATA1 (31). ZnF motifs of GATA factor are highly conserved in different organisms, and we also found a conserved GATA-box in the promoter region of FDH homologs from different species (Fig. S6). Consistent with the observations that GATA factors and FDHs play key roles in nitrogen metabolism among many different fungal species (9–11, 14, 15, 17), our results reveal a possible mechanism by which SsNsd1 regulates *Ssfdh1* and functionally joins with SsFdh1 to repress further transcription of *Ssfdh1* (Fig. S7), ensuring proper accumulation of FDH in fungi nucleus, where it could help maintain NO homeostasis around the chromatin region.

10.1128/mSystems.00397-19.6 FIG S6 Conservation of GATA factors, FDH homologs (FDHs), and the GATA-box in the promoter region of FDHs indicated a potentially wide-ranging role for GATA factors in regulating FDHs. (A) Sequence alignment of GATA binding domains among the GATA factors from different species using ClustalW. Panel A shows a Weblogo presentation of conserved Cys residues after alignment of zinc finger motifs. The overall height of the stack indicates the sequence conservation at that position. (B) Sequence alignment of FDHs from different species using ClustalW. Cys 44 and 173 are indicated by red arrow. (C) The putative GATA-box elements in promoters of *Ssfdh1* orthologs from different organisms. Download FIG S6, TIF file, 1.9 MB. Copyright © 2019 Zhu et al. 2019 Zhu et al. This content is distributed under the terms of the Creative Commons Attribution 4.0 International license.

10.1128/mSystems.00397-19.7 FIG S7 Working model of SsNsd1 regulation and interaction with SsFdh1 in S. sclerotiorum. A proposed model is shown that illustrates that SsNsd1 can positively regulate SsFdh1 by directly binding to the upstream GATA-box in the nucleus. In the cytoplasm, nucleus-localized SsNsd1 translocates SsFdh1 to the nucleus through a stable SsNsd1-SsFdh1 interaction. Finally, the interaction between SsNsd1 and SsFdh1 prevents SsNsd1 from directly binding GATA-box sequences and regulates SsFdh1 and other target genes which influence the development and pathogenicity of S. sclerotiorum. Download FIG S7, TIF file, 0.5 MB. Copyright © 2019 Zhu et al. 2019 Zhu et al. This content is distributed under the terms of the Creative Commons Attribution 4.0 International license.

## MATERIALS AND METHODS

### Fungal strains, plants, and cultural conditions.

Fungal cultures of wild-type (WT) Sclerotinia sclerotiorum isolate 1980 (UF-70) (2) were grown on potato dextrose agar (PDA) at room temperature. Transformants were cultured on PDA medium with 100 μg ml^−1^ hygromycin B (Roche, Indianapolis, IN, USA) or 100 μg ml^−1^ G418 (Sigma-Aldrich, Shanghai, China). Dry stocks of hyphae were maintained at –20°C. Escherichia coli strain DH5α (TaKaRa, Dalian, China) was used to propagate plasmids, and the Agrobacterium tumefaciens EHA105 strain was used for the fungal transformation (45). Sclerotia generated on PDA plates were gathered, air-dried, and stored at –20°C.

Arabidopsis thaliana Col-0 and Nicotiana benthamiana were grown under greenhouse conditions at 21 to 23°C under conditions of a 16-h light/8-h dark cycle with 60% relative humidity (46). Common beans (Phaseolus vulgaris) and tomato (Solanum lycopersicum) were grown under fluorescent lighting in a temperature range of 22 to 25°C in the laboratory. The seeds were planted in a potting soil mix (vermiculite; humus = 1:2).

### Yeast two-hybrid (Y2H) assay and sequence analysis of SsFdh1.

A Matchmaker Gold Y2H system (Clontech, CA, USA) was used to screen for interactors as well as for identification of protein-protein interactions. The bait protein SsNsd1 was used to search against a full-length cDNA library of S. sclerotiorum which was constructed with cDNA from fresh WT mycelial tissues according to the instructions provided for the Matchmaker GAL4-based system. SsFdh1 (SS1G_10135) was identified as one of SsNsd1-interacting proteins. For protein-protein interaction assay, the coding sequences of *Ssnsd1* and *Ssfdh1* were inserted into yeast vectors pGBKT7 and pGADT7, respectively. The Y2H assay was performed according to the standard operational procedures (47).

SmartBLAST was used to search for homologs and to localize highly conserved domains (48). Multiple alignments of protein sequences were performed using BioEdit version 7.0.5 (Tom Hall, CA, USA). The 3D structural modeling of SsFdh1 and SsNsd1 protein was performed on SWISSMODEL (http://www.expasy.ch). The structural model of SsFdh1 and SsNsd1 was visualized with the PyMOL program (http://www.pymol.org/pymol). Manual model building and experiments involving interactions of protein were performed manually using RasMol 2.7.5 (http://www.openrasmol.org/). Transcription factor binding sites in a 2-kb *Ssfdh1* promoter region were determined with LASAGNA-Search 2.0 (http://biogrid-lasagna.engr.uconn.edu/lasagna_search/).

### Gene replacement and complementation.

All the primers used in this study are listed in Table S1 in the supplemental material. The *Ssfdh1* gene was deleted by double homologous recombination. The 5′ region (∼0.9 kb) and the 3′ region (∼1.0 kb) of the *Ssfdh1* gene were amplified from the genomic DNA (gDNA) of WT S. sclerotiorum and were cloned into the pXEH vector (46) to generate a pXEH-R-L construct containing a hygromycin phosphotransferase (hph) cassette driven by a *trpC* promoter. Hygromycin-resistant transformants obtained by protoplast transformation (25), namely, knockout (KO) strains, were verified by PCR. All of the transformants were purified by three rounds of hyphal-tip transfer.

10.1128/mSystems.00397-19.8 TABLE S1 Primer list. Download Table S1, DOCX file, 0.02 MB. Copyright © 2019 Zhu et al. 2019 Zhu et al. This content is distributed under the terms of the Creative Commons Attribution 4.0 International license.

For genetic complementation of *Ssfdh1* KOs, a genomic region containing the full-length fragment of *Ssfdh1* (∼3.9 kb), including 944-bp 5′ upstream and 1,027-bp 3′ downstream of the coding sequence, was amplified from the WT S. sclerotiorum gDNA. This fragment was then cloned into pD-NEO1 carrying the strong promoter *trpC*, which was constructed from vectors pSilent-Dual1 and pD-NAT1 (46). KO strain *Ssfdh1*-KO.1 was used for genetic complementation. The complementation transformants were screened on PDA medium with 100 μg ml^−1^ G418 and then verified by PCR.

To identify mutants, gDNA was extracted from the WT, the KOs, and a complementation strain (C-22) for Southern blot analysis. Extracted gDNA was digested with XbaI and BamHI (TaKaRa, Dalian, China), separated on 0.8% agarose gel by electrophoresis, and transferred to a nylon membrane (Hybond-N^+^; GE Healthcare). Probe 1 was the PCR-amplified product of an 878-bp fragment from the *Ssfdh1* gene. Probe 2 was a 768-bp fragment amplified from the *hph* gene. Probe 3 was a 672-bp fragment amplified from a G418 resistance gene.

### Morphological characterization.

Agar-mycelium plugs from the WT strain, KOs, and strain C-22 were placed on PDA medium at 25°C. The diameters of colony on PDA medium were measured at different time points to evaluate the radial mycelial growth. For quantification of compound appressoria, fresh mycelial plugs were inoculated on paraffin film and then incubated under suitable humidity conditions for 3 days. For morphological observation of compound appressoria, a fresh PDA-colonized agar plug (5 mm in diameter) was placed on a glass slide (Sail brand) and was placed in a box at room temperature for 24 h. Compound appressoria were observed with an optical microscope (BA310Met-T; Ted Pella, Xiamen, China). Mature sclerotia of each strain were collected from smashed potato agar (SPA) medium and photographed at 7 days after inoculation (DAI) using a Sony SELP1650 digital camera (Sony, Tokyo, Japan). Sclerotium development was determined by measuring dry weight and size. For induction of apothecia, mature sclerotia were cultured in a 15°C incubator at 70% humidity and with constant illumination. Germination rates (corresponding to the proportion of sclerotia producing apothecia) were calculated after 60 days as described previously (6).

### Enzyme activity assay.

UV spectroscopy was used to analyze enzyme activities (38). Different strains were cultured in liquid potato dextrose medium (24 h shake/24 h static, normal illumination) at room temperature for 7 days. The supernatant was removed by centrifuging 25-ml cultures at 7,000 rpm and 4°C. To obtain intracellular crude enzymes from hypha, mycelia were added with 50 ml 0.05 M potassium phosphate buffer solution (pH 7.5) and washed twice; the same buffer solution was then added to the mycelium pellet (1.5:1 ratio [vol/vol]) to suspend the pellet. The mycelium cells were then crushed by the use of an ultrasonic cell crusher (2 horn; 22% power; ultrasound 2 s, stop 3 s) on ice for 15 min and were centrifuged at 15,000 rpm for 30 min at 4°C to remove cell debris. Enzyme activity reactions were performed by combining 1 ml of crude extract from different genotypes with 1.5 ml various reaction buffers, such as 0.1 mM formaldehyde, methyl alcohol, or methanoic acid in 0.05 M potassium phosphate buffer solution (pH 7.5) at 37°C for 1 h. Radiation absorbance at 540 nm was determined by the use of a UV spectrometer. Enzyme activity was defined using the following equation: (units per milliliter) ＝ [(sample level – control level) × Dr]/(*K* × *V* × *T*), where *K* represents the standard curve slope, Dr represents the enzyme dilution ratio, *V* represents the enzyme solution volume, and *T* represents the reaction time (h) (49).

### Stress adaptation assays.

To determine if SsFdh1 is involved in formaldehyde metabolism and nitrosative stress in S. sclerotiorum, exogenous formaldehyde and nitric oxide (NO) sensitivity assays of the KOs were performed (14). For formaldehyde sensitivity assay, PDA medium was supplemented with 0 or 0.3 mM formaldehyde. For NO stress assay, PDA medium was supplemented with 0.25 or 0.5 mM SNP (sodium nitroprusside) as a NO donor. Colony diameters of mycelia were measured at 3 DAI.

Stress adaptation assays were also performed on PDA medium supplemented with an osmotic stress agent (KCl, NaCl, or sorbitol), a cell wall-damaging agent (SDS), and an oxidative stress agent (H_2_O_2_) as previously described (38, 45). Agar plugs with colonized WT, KO, and C-22 strains were inoculated on PDA plates containing 1 M KCl, 1 M NaCl, 1 M sorbitol, 0.02% SDS, and various concentrations of H_2_O_2_. Mycelium colony diameters were then measured at 3 DAI.

### Pathogenicity assay.

To assess fungal pathogenicity, healthy or wounded common bean leaves or fully expanded tomato leaves were inoculated with fresh PDA-colonized agar plugs of different strains and placed into an incubator for 3 to 4 days (50). Each strain was used to inoculate three leaflets from different plants, and the pathogenicity experiments were repeated three times on the different days. Infection symptoms were recorded, and the lesion areas were determined by ImageJ (https://imagej.nih.gov/ij/).

### Gene expression level analysis by qRT-PCR.

To profile the expression of *Ssnsd1* and *Ssfdh1* in different developmental stages, tissues of hypha, sclerotia (S1 to S6), and apothecia (A1 to A6) were collected for RNA preparation and qRT-PCR (36). The qRT-PCR conditions were as described previously (46) with a histone H3 gene (SS1G_09608) as an internal reference, and the relative levels of gene expression were analyzed using the threshold cycle (2^-ΔΔ^*^CT^*) method (6). Each qRT-PCR experiment was replicated at least three times. The expression levels of sclerotium development-related genes and infection cushion generation-related genes in the WT and Δ*Ssfdh1* strains were also determined using qRT-PCR as described above. qRT-PCR was also used to evaluate *Ssfdh1* and *Ssnsd1* expression levels after formaldehyde, methyl alcohol, and methanoic acid treatment. Briefly, the S. sclerotiorum WT strain was cultured in liquid potato dextrose medium at 28°C for 2 days with shaking, and then the samples were treated with 0.3 M formaldehyde, methyl alcohol, or methanoic acid for 2 h.

### Bimolecular fluorescence complementation (BiFC) assay.

BiFC assay was used in an *Arabidopsis* protoplast system to confirm protein interaction (51). SsNsd1 and SsFdh1 were fused with separate regions of yellow fluorescent protein (YFP) (52) to generate YFP-N-SsNsd1 and YFP-C-SsFdh1 for *Arabidopsis* protoplast transfection (53). The fluorescent signal and localization of fusion proteins were detected using an inverted fluorescence microscope (Eclipse Ts2R; Nikon, NY, USA) (emission, 514 nm). The nucleus was visualized using the fluorescent dye DAPI (4′,6-diamidino-2-phenylindole).

### Coimmunoprecipitation assay.

Full-length coding regions of *Ssnsd1* and *Ssfdh1* were cloned into pCG-1301-GFP and pCHF-3301-3xFLAG vectors (54) to generate pCG-*Ssnsd1*-GFP and pCHF-*Ssfdh1***-**3×FLAG constructs. *Agrobacterium* carrying different plasmids was coinfiltrated into N. benthamiana leaves, and tissue samples were harvested 2 days after infiltration. The leaf tissues were ground into fine powder in liquid nitrogen, and the total protein was extracted with protein extraction buffer (20 mM Tris-Cl [pH 7.5], 150 mM NaCl, 20% glycerol, 5 mM EDTA, 2 mM dithiothreitol [DTT], 1% protease inhibitors, 0.5% NP-40). The protein extract was then subjected to anti-GFP immunoprecipitation. After 1 h of end-to-end rotation binding at 4°C, the GFP resins were precipitated by centrifugation at 500 × *g* for 30 s and the resins were washed 4 times with wash buffer (20 mM Tris-Cl [pH 7.5], 150 mM NaCl, 20% glycerol, 2 mM DTT, 1% protease inhibitors, 0.2% NP-40). The presence of each protein was determined by immunoblotting performed with either anti-FLAG antibody or anti-GFP antibody (55).

### Site-directed mutagenesis.

The selected cysteine residues of SsFdh1 and SsNsd1 were substituted by alanine by the use of a fast mutagenesis system (Tran, Beijing, China). The double point mutants *Ssfdh1*^C44/C173^, *Ssnsd1*^C331/C334^, and *Ssnsd1*^C353/C356^ were constructed successively (Fig. S7). The sequences of *Ssfdh1* and *Ssnsd1* point mutants were cloned into the respective vectors for Y2H and BiFC analysis. They were also then cloned into pNDB-OCT (56) and pCB-GFP (57) vectors for functional analysis.

### Electrophoretic mobility shift assay (EMSA).

The coding regions of *Ssnsd1*, *Ssnsd1*^C331/C334^, *Ssfdh1*, and *Ssfdh1*^C44/C173^ were cloned into pET28a vector for protein expression in E. coli BL21, and the recombinant proteins were purified using nickel-nitrilotriacetic acid (Ni-NTA). A 30-bp DNA probe containing the GATA sequences from the *Ssfdh1* promoter was labeled with or without digoxigenin-11-ddUTP (DIG-11-ddUTP) at the 3′ end (see Data Set S2 in the supplemental material). We used a DIG gel shift kit, 2nd generation (Roche, USA), to perform the EMSA. Reaction mixtures containing purified protein and probes were incubated for 20 min at 25°C. The samples were analyzed by 5% polyacrylamide gel electrophoresis in 1× Tris-borate (TB) buffer containing 90 mM TB and separated by electrophoresis at 120 V for 2 h. GATA TF-specific commercial antibodies (58, 59) (Santa Cruz, CA, USA) used for supershift assays were added to the reaction mixtures before incubation with the labeled probe was performed. Signals were detected using chemiluminescent substrate in the kit according to the manufacturer’s instructions (60).

### Fluorescent protein localization in S. sclerotiorum.

To detect the subcellular localization of SsNsd1, the cDNA fragment of *Ssnsd1* was cloned into pCB-GFP vector (57). The resulting construct (pCB-*Ssnsd1*-GFP) was transformed into a S. sclerotiorum WT protoplast, and the transformants were identified with 50 μg ml^−1^ chlorimuron-ethyl on PDA medium. Similarly, the cDNA region of *Ssfdh1* was cloned into pNDB-OCT to generate a pNDB-*Ssfdh1*-mCherry construct. The resulting plasmids were transformed into the WT or ***Δ****Ssnsd1* protoplast, and the transformants were screened on the PDA medium with 50 μg ml^−1^ bleomycin (61). For the colocalization assay, SsNsd1 and SsNsd1^C331/C334^ were cloned into pCB-GFP vector, the resulting constructs were used to transform protoplasts of ***Δ****Ssnsd1* containing *Ssfdh1*-mCherry, and the transformants were screened on PDA with 50 μg ml^−1^ chlorimuron-ethyl (62). Fluorescence localization was observed with a fluorescence inverted microscope (emission: GFP, 488 nm; mCherry, 587 nm) with nuclei stained with DAPI.
