# Supplementary figures and images for: The Formaldehyde Dehydrogenase SsFdh1 Is Regulated by and Functionally Cooperates with the GATA Transcription Factor SsNsd1 in Sclerotinia sclerotiorum
Source: mSystems. 2019 Sep 10;4(5):e00397-19. doi: 10.1128/mSystems.00397-19 (PMC6739101; doi:10.1128/mSystems.00397-19)

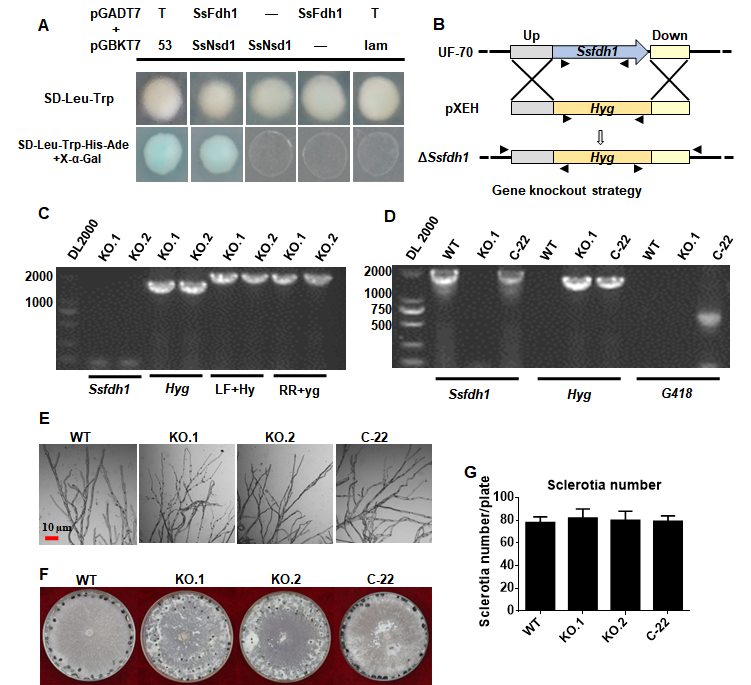

Supplement: FIG S1 [file mSystems.00397-19-sf001.tif]

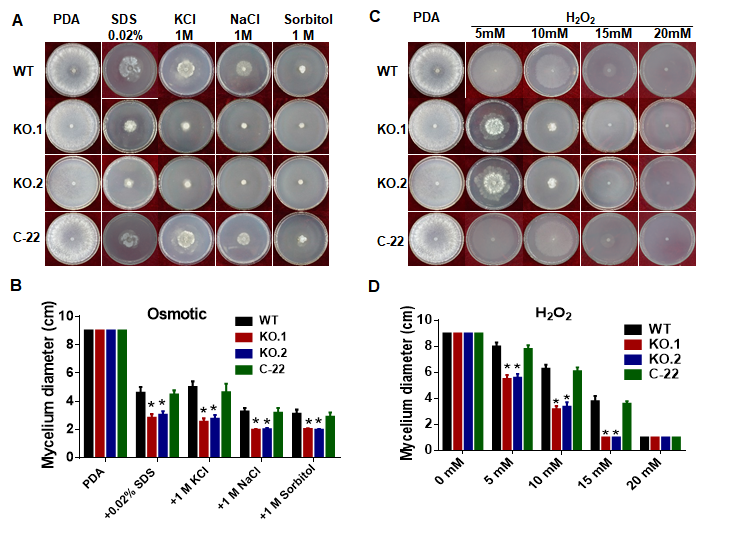

Supplement: FIG S2 [file mSystems.00397-19-sf002.tif]

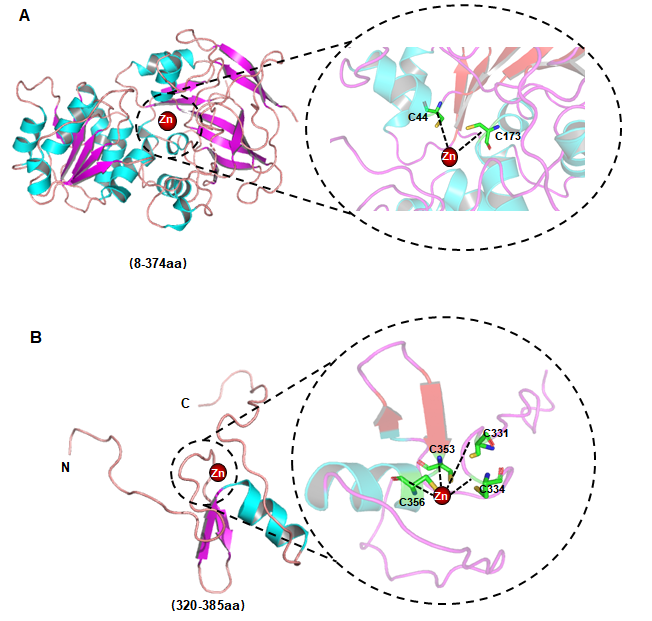

Supplement: FIG S3 [file mSystems.00397-19-sf003.tif]

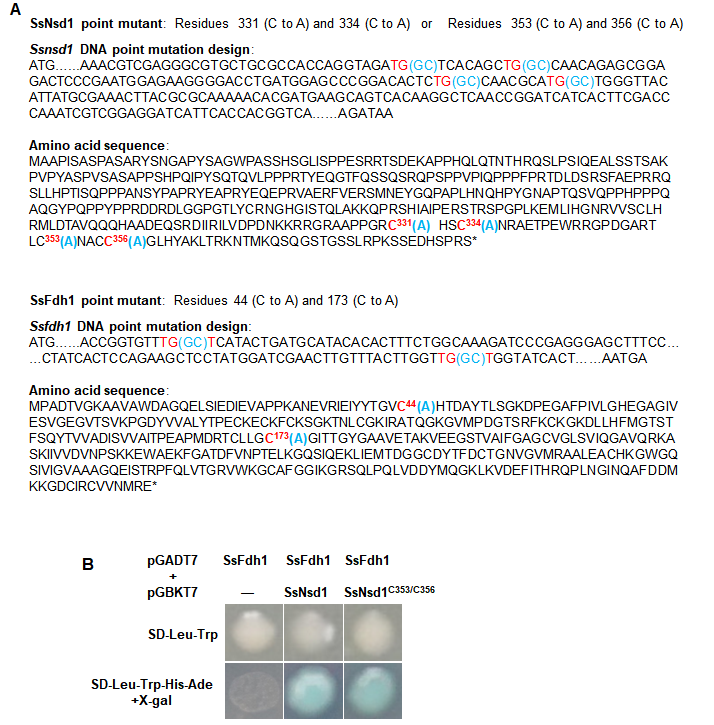

Supplement: FIG S4 [file mSystems.00397-19-sf004.tif]

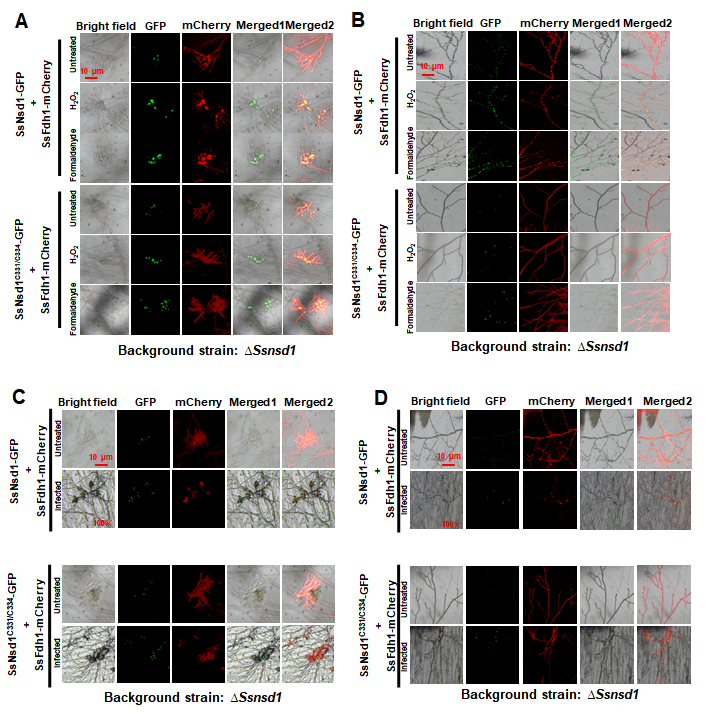

Supplement: FIG S5 [file mSystems.00397-19-sf005.tif]

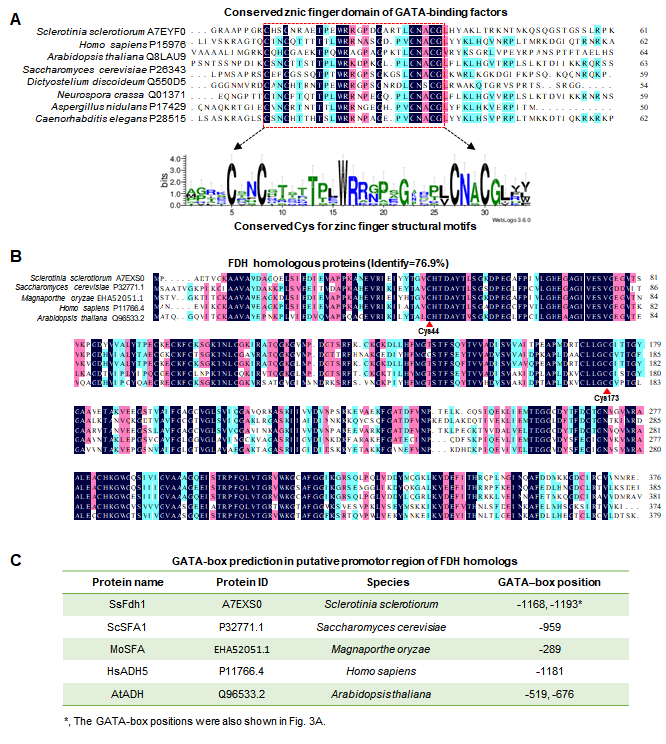

Supplement: FIG S6 [file mSystems.00397-19-sf006.tif]

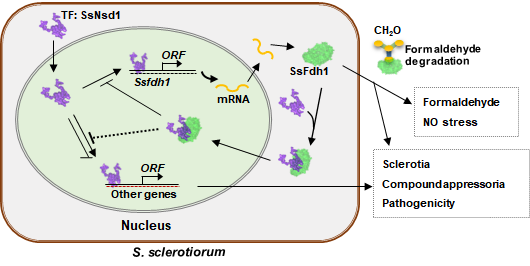

Supplement: FIG S7 [file mSystems.00397-19-sf007.tif]
