# Supplementary material for: The Formaldehyde Dehydrogenase SsFdh1 Is Regulated by and Functionally Cooperates with the GATA Transcription Factor SsNsd1 in Sclerotinia sclerotiorum
Source: mSystems. 2019 Sep 10;4(5):e00397-19. doi: 10.1128/mSystems.00397-19 (PMC6739101; doi:10.1128/mSystems.00397-19)
Supplement: DATA SET S1 [file mSystems.00397-19-sd001.docx]

| **Supplementary Data 1. A list of putative SsNsd1-interacting proteins identified by yeast two-hybrid screens. (*** represents no homologs and no predicted function)**.** | | |
| --- | --- | --- |
| Proteins | Homologs in | Putative functions |
| SS1G_00872 | TDH2 | There are three genes for G3PDH in yeast;glyceraldehyde-3-phosphate dehydrogenase (phosphorylating) TDH2. |
| SS1G_10135 | ADH5 | Members identified as glutathione-dependent formaldehyde dehydrogenase(FDH), a member of the zinc dependent/medium chain alcohol dehydrogenase family. FDH converts formaldehyde and NAD(P) to formate and NAD(P)H. |
|  | SOR1 | Acts synergistically with sop-2 to maintain the transcriptionally repressive state of homeotic genes throughout development. Not required to initiate repression, but to maintain it during later stages of development. Also required to repress expression of other genes. Binds RNA in a sequence-independent manner. |
| SS1G_12932 | * | * |
| SS1G_14138 | YSF3 | Involved in pre-mRNA splicing. Required for the SF3b integrity and prespliceosome assembly. |
| SS1G_02257 | SAL1 | Calcium-dependent mitochondrial solute carrier. |
|  | FLX1 | Transport of FAD from the cytosol to the mitochondrial matrix. |
|  | TPC1 | Mitochondrial transporter that mediates uptake of thiamine pyrophosphate (ThPP) into mitochondria. |
|  | AAC1 | Catalyzes the exchange of ADP and ATP across the mitochondrial inner membrane. |
|  | AGC1 | Calcium-dependent mitochondrial aspartate and glutamate carrier. Transport of glutamate in mitochondria is required for mitochondrial transamination reactions and ornithine synthesis. Plays also a role in malate-aspartate NADH shuttle, which is critical for growth on acetate and fatty acids. |
| SS1G_04744 | * | * |
| SS1G_14065 | * | * |
| SS1G_00934 | * | * |
| SS1G_07798 | TDH1 | Cytochrome c oxidase is the component of the respiratory chain that catalyzes the reduction of oxygen to water. Subunits 1-3 form the functional core of the enzyme complex. CO I is the catalytic subunit of the enzyme. Electrons originating in cytochrome c are transferred via the copper A center of subunit 2 and heme A of subunit 1 to the bimetallic center formed by heme A3 and copper B. |
| SS1G_12522 | * | * |
| SS1G_05105 | MCM1 | Transcription factor required for the efficient replication of minichromosomes and the transcriptional regulation of early cell cycle genes. Activates transcription of ECB-dependent genes during the G1/M phase. Genes that contain a ECB (early cell box) element in their transcription regulatory region are transcribed only during G1/M phases. Interacts with the alpha-2 repressor or with the alpha-1 activator thereby regulating the expression of mating-type-specific genes. With ARG80, ARG81 and ARG82, coordinates the expression of arginine anabolic and catabolic genes in response to arginine. |
| SS1G_00905 | * | * |
| SS1G_07183 | * | * |
| SS1G_07033 | PIP2 | The PIP2-OAF1 heterodimer acts as a transcriptional activator to induce the transcription of genes encoding proteins involved in fatty acid beta-oxidation, a response called oleic acid induction, when cells grow on fatty acids as sole carbon source. Recognizes and binds to the oleate response element (ORE) (or peroxisome box), two inverted CGG triplets spaced by 14 to 18 intervening nucleotides, in the promoter region of a number of genes (such as CTA1, FOX1 to FOX3, FAA2, PAS8, PAS10, etc.) for peroxisomal proteins. Activity is inhibited by OAF1 under non-inducing conditions. Activity is repressed by glucose. |
| SS1G_07944 | CDC14 | Protein phosphatase which antagonizes mitotic cyclin-dependent kinase CDC28, the inactivation of which is essential for exit from mitosis. To access its substrates, is released from nucleolar sequestration during mitosis. Plays an essential in coordinating the nuclear division cycle with cytokinesis through the cytokinesis checkpoint. Involved in chromosome segregation, where it is required for meiosis I spindle dissambly as well as for establishing two consecutive chromosome segregation phases. |
|  | PTP2 | Major phosphatase responsible with PTP3 for tyrosine dephosphorylation of MAP kinase HOG1 to inactivate its activity. May also be involved in the regulation of MAP kinase FUS3. May be implicated in the ubiquitin-mediated protein degradation. |
| SS1G_03736 | PMA1 | The plasma membrane ATPase of plants and fungi is a hydrogen ion pump. The proton gradient it generates drives the active transport of nutrients by H^+^-symport. The resulting external acidification and/or internal alkinization may mediate growth responses. |
|  | PMR1 | This magnesium-dependent enzyme catalyzes the hydrolysis of ATP coupled with the transport of calcium. Has a role in the secretory pathway. |
|  | ENA1 | Has phosphatase activity in vitro. Involved in the response to sodium and lithium ion stress (but not to potassium or sorbitol stress) by inducing transcription of the sodium pump ENA1/PMR2. Acts through a calcineurin-independent pathway and is functionally redundant with PSR2. Also involved in the general stress response; acts together with WHI2 to activate stress response element (STRE)-mediated gene expression, possibly through dephosphorylation of MSN2 |
|  | PCA1 | Cadmium transporting P-type ATPase which plays a critical role in cadmium resistance by extruding intracellular cadmium. Capable of high affinity copper ion binding, but not active copper ion transport. May play a role in copper resistance by chelating and sequestering copper ions. |
| SS1G_03803 | * | * |
| SS1G_01226 | * | * |
| SS1G_08945 | * | * |
| SS1G_05105 | * | * |
| SS1G_14286 | SNT2 | [Transcriptional regulator that, together with ECM5, recruits histone deacetylase RPD3 to a small number of promoters of stress-response genes in response to oxidative stress (PubMed:23878396). Probable ubiquitin-protein ligase involved in the degradation-related ubiquitination of histones. Contributes to the post-translational regulation of histone protein levels by polyubiquitination of excess histones for subsequent degradation](https://www.uniprot.org/citations/23878396) |
| SS1G_00632 | CDC34 | Catalyzes the covalent attachment of ubiquitin to other proteins. Capable, in vitro, to ubiquitinate histone H2A. Mediates the initiation of DNA replication (transition of G1 to S phase in cell cycle). Essential component of the E3 ubiquitin ligase complex SCF (SKP1-CUL1-F-box protein), which mediates the ubiquitination and subsequent proteasomal degradation of target proteins. Involved in the regulation of methionine biosynthesis genes and in the degradation of CDC6 together with CDC4 and CDC53. |
|  | UBC7 | Catalyzes the covalent attachment of ubiquitin to other proteins. Functions in degradation of misfolded or regulated proteins localized in the endoplasmic reticulum (ER) lumen or membrane via the ubiquitin-proteasome system. Cognate E2 conjugating enzyme for the DOA10 ubiquitin ligase complex, which is part of the ERAD-C pathway responsible for the rapid degradation of membrane proteins with misfolded cytoplasmic domains, and of the HRD1 ubiquitin ligase complex, which is part of the ERAD-L and ERAD-M pathways responsible for the rapid degradation of soluble lumenal and membrane proteins with misfolded lumenal domains (ERAD-L), or ER-membrane proteins with misfolded transmembrane domains (ERAD-M). Involved in resistance to cadmium poisoning. |
|  | RAD6 | Catalyzes the covalent attachment of ubiquitin to other proteins. In association with the E3 enzyme BRE1 and LGE1, it plays a role in transcription regulation by catalyzing the monoubiquitination of histone H2B to form H2BK123ub1. H2BK123ub1 gives a specific tag for epigenetic transcriptional activation, elongation by RNA polymerase II, telomeric silencing, and is also a prerequisite for H3K4me and H3K79me formation. In association with the E3 enzyme RAD18, it catalyzes the monoubiquitination of POL30 'Lys-164', involved in postreplication repair of UV-damaged DNA. The RAD6/UBC2-RAD18 complex is also involved in prevention of spontaneous mutations caused by 7,8-dihydro-8-oxoguanine. In association with the E3 enzyme UBR1, is involved in N-end rule-dependent protein degradation. Also involved in sporulation. |
| SS1G_07837 | * | * |
| SS1G_13013 | * | * |
| SS1G_06590 | CBK1 | Protein kinase that seems to play a role in the regulation of cell morphogenesis and proliferation. |
| SS1G_05913 | * | * |
| SS1G_07889 | KEX2 | Processing of precursors of alpha-factors and killer toxin. |
| SS1G_07642 | DHH1 | ATP-dependent RNA helicase involved in mRNA turnover, and more specifically in mRNA decapping by activating the decapping enzyme DCP1 . Is involved in G1/S DNA-damage checkpoint recovery, probably through the regulation of the translational status of a subset of mRNAs . May also have a role in translation and mRNA nuclear export. Required for sporulation. Blocks autophagy in nutrient-rich conditions by, at least partly, binding and repressing the expression of a set of ATG genes, including ATG3, ATG7, ATG8, ATG19, ATG20, ATG22 and SNX4/ATG24 . |
|  | PBS2 | Kinase involved in a signal transduction pathway that is activated by changes in the osmolarity of the extracellular environment. Seems to phosphorylate HOG1 on a tyrosine residue. |
| SS1G_00036 | * | * |
| SS1G_10588 | * | * |
| SS1G_03527 | * | * |
| SS1G_11231 | ATG1 | Serine/threonine protein kinase involved in the cytoplasm to vacuole transport (Cvt) and found to be essential in autophagy, where it is required for the formation of autophagosomes. Involved in the clearance of protein aggregates which cannot be efficiently cleared by the proteasome. |
|  | KIN3 | This protein is probably a serine/threonine protein kinase. |
| SS1G_00095 | * | * |
| SS1G_06834 | * | * |
| SS1G_12468 | TPK2 | Voltage-independent, large conductance and potassium-selective tonoplast ion channel. Regulated by cytoplasmic calcium and pH. Does not mediate slow-vacuolar (SV) ionic currents, but essential to establish VK currents. Has some permeability for Rb^+^ and NH_4_^+^, but none for Na^+^, Cs^+^ or Li^+^. Involved in intracellular K^+^ redistribution and/or K^+^ retranslocation between different tissues. |
| SS1G_12239 | PDR5 | Active efflux of weakly charged organic compounds of 90 cubic Angstroms to 300 cubic Angstroms surface volume. Confers resistance to numerous chemicals including cycloheximide, sulfomethuron methyl, steroids, antiseptics, antibiotics, anticancer, herbicides, mycotoxins, insecticides, ionophores, alkaloids, flavonoids, phenothiazines, organotin compounds, carbazoles, lysosomotropic aminoesters, detergents, rhodamines and other fluorophores, azoles and other antifungals. Exhibits nucleoside triphosphatase activity. |
| SS1G_02740 | * | * |
| SS1G_02211 | BDH2 | Catalyzes the irreversible reduction of 2,3-butanediol to (S)-acetoin in the presence of NADH. |
| SS1G_08742 | GPI3 | Catalytic subunit in the complex catalyzing the transfer of N-acetylglucosamine from UDP-N-acetylglucosamine to phosphatidylinositol, the first step of GPI biosynthesis. |
| SS1G_02128 | OPI9 | Partially overlaps VRP1. Disruption phenotypes caused by deletion of this gene may also be a result of a defect in its overlapping gene. |
