# Supplementary material for: The Formaldehyde Dehydrogenase SsFdh1 Is Regulated by and Functionally Cooperates with the GATA Transcription Factor SsNsd1 in Sclerotinia sclerotiorum
Source: mSystems. 2019 Sep 10;4(5):e00397-19. doi: 10.1128/mSystems.00397-19 (PMC6739101; doi:10.1128/mSystems.00397-19)
Supplement: DATA SET S2 [file mSystems.00397-19-sd002.docx]

**Supplementary Data 2. *Ssfdh1* coding sequence and its putative promoter region (coding sequences are colored with green and promoter region is colored with blue).**

AGTTATAAACCATCTCGAGAGCCTAGAGCACTTCCACGTGATCTAGACGGCAAACCTCGTCACAATTCTATATAAATTGAAAATCGTAATCTCAATCATTTATATTTCAATCAATAAAATCGCGAAAAATCCGAAATTTTGAGAATCAATGAATAATTATATAATAAATCTAGAATTAAAATTTCAATTGATTTCATTCATTGGTTGTTGAAATATTAATAGGTGAAAATCATGATTTTTTGTTACAACGGCTATCTTCTATGCCGCGTCACATGACTAAACCAAGGTAACGTGATTAAAGTCACGTTATGGACCGCTGAGGAACGCTGGCTGTCCAGGACCGCGCAGCGGTCCTGACTAGCCATAGGCATGAAGGTCTATATAATCAAGATGGATTCCCTTTGTATAGAGCTTCGTGCTCAAGATCAATCACTTAGTATACTCTACTATTAGTTACGAATTGCATATCATTATTGTCATTACTTTAAGTCCCTGCTTGACGCCTAGTTATAAACCACCTCGAGAGCCTAGAGCACTTCCACGTGATCTAGACGGCAAACCTCGTAACATTTTTGGTGTGAAAGGGTAGCCGAAAAGTGGGGTACTAGACTTAATTGGGGAATAGGAGTGAGGACTCAAGAGACTCTCTTGAGGAGAGGAGGAGAGAGATAGAGTTTCCGAATTGCGAGGAGTTGATTCGGTATTTCTTTTGAGAGAAGAGGGGCGAGGTGGGGTTTGGTTGTGTGTAAATCAGGCCTATTTTCTCTGGTGTATACGGGTTTCTGGTGTATTGAGGTTGTTGTAGCTTTGTTTTCATTGTGGGCTTATTATATGATCAATCTATCAAAATTCTCAAGGTGGGGATATAACAGGGTTTATCTATATTTATCAAAAGTGTTGTATTAATAAATAATACTTGTATATATTGGAATCATGAAATTGTTGTCTGATGTAGATAAAATACCGAAATTTCCGGTATAATCATAAGGTTACAAAATAATGAAAGCATACGAAATATAATCGGTATAGGCGGGATTCCGGTATAATAATGTCTGGTATAGCGAGGTTCTACTGTATGTGTGCAGAGGATCTTGCCGGGATGGAGGGAGGGGGATAGAGAGGGGAAGAGGGAGAGGAAGAGGGGTAGATTGAGTGTTAGAATTTATACAGGAATTGTTTGTTGTATTATATAGTACCATAGTAGGTTCTTAATGATTCGTGATACCATAGAGGATTTATCAACCGCACTCAGTCATCCTATCTCTGATTCACATGACGAGAATCTGATGCGGTTCACTATGAAGTAAATATTACTCAATCTAGATAAAAGATCCCGAAGTGCGGGGTTTATGGTGTAGGATTGGGAAGTGTTTTATATGTGGAATCGTGGATAGATGGATGAATGCCGATGTGCTCCGATTTCATTGTTAAGCCTTTTGGTATACGCAATAATGGTGTGTATTTGGTAGAAGGAACTTTCTTGGTATTTATTTAAGAGGGATGTTGGTAGGTAGAAGA

Two GATA-boxes were highlighted in yellow

TGTCTTATCATGATGATGGATGA**TTGTCGATTGATAATTGATAATTGATACTT**GCATTCCCAGCAATCT

The EMSA probe was highlighted in red

CGCCGTGTGAGTCAAATATCGATATCTTCATCTTCATCCACAAGTACATACAAATTAATACAAGTACCGTATAGTTTTATCTCTAGCTTTACAATACCCCATTAGGAAAACCTCAGGAGTGTACAATTAATTAATTACCTTACTTACGTACCGAGTTCTGCGAATTTCACAAGAGGTGGGGTATCCAAAGTAAAAGCTTGCCCCTCACTACAACTTGTCCGAAAGAAGTCCGCTCCAACCTCCTTTACTAATATATTGCTACACGGTACTTCATACTTGATTTCTTTCGAAGCATCTACAACGTAACTTTTTGTACACCTTGAGAGCTTGCTTTATACCTTGATAGCTTTTTTTGTCAAGCGGACGAATTGTGTTACGGAGAGAACAGAAGAACGAAAAAGAGAATAGGAAACATGCCGGCTGATACTGTTGGGAAGGTAGGTTCGATTTTATTTGGATTGAATTGGTGGGGTGAGGTGGGATGATGAGAGTGTGGCAGAACTGTTTACGATTGATTGATGTATATTTGCGGATTGGCCTCATGGACTTGTGGAAACATATGCTAATGAGTGGTTTATTATAGACCATTACCTGCAAGGTTAGTTCGACAATTCTATATTCTACCTATTAGACTATACGATTCTGCTATATCATAGCACTCGGTGTACTTGGGAGGGCTTTTGTGAAATTTGCGGATGCAGTGGCAACTCTGCGGATGGCTCGATAGCTTTGCGTAGAGTATAACAGTTCTGCGAAGCTTCAATCCCCTAGACCCAAATCCGCAGTTTTCTCAAACACTCCCCAGCCCACTACATTCATCCCATATATTAAACATCACTCTTGGAATGAATAGTGAACCGGGGTAGGCAGAAGAAGAAACAAAAGCTGATGAAATATATAGGCAGCAGTTGCCTGGGACGCAGGTCAAGAACTCAGTATTGAGGATATTGAGGTTGCTCCTCCCAAGGCAAATGAAGTGAGAATTGAGATTTATTATACCGGTGTTTGTCATACTGGTAGGTTTTAATGTTTCTTCTTGTGTGGTGAAGCTTGGAGGTGGTGAGAAGGTGAGGTAGGAGAGCGGGGTTGTGATTCGGAAACGGATGATGGGTGTAGTACGCATAGGGTCGTACAAAGATGATGGAGCATGAGAAAATTGATTCAAGGATGAATGCTAACAAATCATCATAATAGATGCATACACACTTTCTGGCAAAGATCCCGAGGGAGCTTTCCCAATTGTTCTTGGACATGAGGGTGCTGGTATTGTTGAATCTGTTGGTGAAGGTGTAACATCTGTTAAACCTGGTGATTATGTTGTTGCTTTATAGTATGTCCTCAGCGTCAATTTTCTTCTACCTCTTGTTATTTATTCTTATTATCTCATCACCATCGCTGTACCTGATACCCTTCTAACTCCATTTACCTCATCCTCTTCTCTACACAACCCACTAACAACTACACAGCACCCCAGAATGTAAGGAATGTAAATTCTGTAAATCTGGTAAGACCAACCTTTGTGGAAAAATTAGAGCAACTCAAGGAAAGGGTGTTATGCCAGATGGTACATCCAGATTCAAGTGCAAGGGTAAAGATCTTCTTCATTTCATGGGAACATCTACCTTTTCTCAATATACTGTCGTTGCCGATATTTCCGTTGTAGCTATCACTCCAGAAGCTCCTATGGATCGAACTTGTTTACTTGGTTGTGGTATCACTACCGGTTACGGAGCTGCTGTCGAAACCGCTAAAGTCGAAGAAGGATCTACTGTTGCTATCTTCGGTGCGGGTTGTGTTGGTCTCAGTGTTATTCAAGGAGCTGTACAAAGGAAAGCTTCAAAGATCATCGTGGTCGATGTTAATCCATCCAAGAAAGAATGGGCCGAGAAATTTGGTGCAACAGATTTCGTTAACCCAACTGAATTGAAGGGTCAAAGTATTCAAGAAAAATTAATCGAGATGACCGATGGTGGTTGTGATTATACTTTTGATTGTACTGGAAATGTTGGAGTTATGAGAGCTGCTTTGGAAGCTTGTCATAAAGGTTGGGGTCAAAGTATTGTTATTGGTGTTGCTGCTGCTGGACAGGAGATTAGTACTAGACGTGAGTTTTTCCCCTCTTTCCACGTTACTTCTCAATCACAGACATAAATAACTAACATAATATTAAACAGCATTCCAACTTGTCACAGGTCGTGTTTGGAAAGGTTGTGCATTTGGTGGTATTAAAGGTCGTTCTCAATTACCTCAATTGGTTGATGATTACATGCAAGGAAAATTAAAGGTTGATGAGTTCATTACTCATAGACAACCTTTGAACGGAATTAACCAAGCATTTGATGATATGAAGAAGGGGGACTGTATTAGGTGTGTGGTTAATATGAGAGAATGA
