# Supplementary material for: The Formaldehyde Dehydrogenase SsFdh1 Is Regulated by and Functionally Cooperates with the GATA Transcription Factor SsNsd1 in Sclerotinia sclerotiorum
Source: mSystems. 2019 Sep 10;4(5):e00397-19. doi: 10.1128/mSystems.00397-19 (PMC6739101; doi:10.1128/mSystems.00397-19)
Supplement: TABLE S1 [file mSystems.00397-19-st001.docx]

**Supplementary Table 1. Primer list.**

| Primers | Sequences 5'-3' | Purpose |
| --- | --- | --- |
| Mut^C44/C173^-1 F | CGCCACCAGGTAGAGCTCACAGCTG | SsFdh1 mutation |
| Mut^C44/C173^-1 R | CAGCTCCCGCACGACGCGGTGGTCCATCTACCG |  |
| Mut^C44/C173^-2 F | TAGAGCTCACAGCGCCAACAGAGCGGA |  |
| Mut^C44/C173^-2 R | GGTGGTCCATCTCGAGTGTCGACCG |  |
| Mut^C331/C334^-1 F | ATTATACCGGTGTTGCTCATACTGATG | SsNsd1 mutation |
| Mut^C331/C334^-1 R | TCTTAACTCTAAATAATATGGCCACAACG |  |
| Mut^C331/C334^-2 F | TTGTTTACTTGGTGCTGGTATCACT |  |
| Mut^C331/C334^-2 R | CCTATGGATCGAACTTGTTTACTTGGTGC |  |
| Mut^C353/C356^-1 F | AGCCCGGACACTCGCCAACGCATGT |  |
| Mut^C353/C356^-1 R | ACCTGATGGAGCCCGGACACTCGC |  |
| Mut^C353/C356^-2 F | CTCGCCAACGCAGCTGGGTTACATTATG |  |
| Mut^C353/C356^-2 R | TGGAGCCCGGACACTCGCCAACGCAGC |  |
| qPCR-Fdh1 F | CTGTTGGGAAGGCAGCAGTT | Profiling *Ssfdh1* and *Ssnsd1* transcripts |
| qPCR-Fdh1 R | TTTGCCTTGGGAGGAGCAAC |  |
| qPCR-Nsd1 F | CCGCCAAGCCTGTTCCGTATG |  |
| qPCR-Nsd1 R | GGAGAAGGTTGCCGTGATTGAGAC |  |
| qPCR-Sssac1 F | CAAGCCGAAGTCCAAGCACCTG | Profiling sclerotia-related gene expression |
| qPCR-Sssac1 R | TCCTTCTGTCTTGACCTCGTAGCC |  |
| qPCR-Sspka1 F | TGAGCGTTCGGTTCTTGCAGATG |  |
| qPCR-Sspka1 R | ATCGTGCTGTGTTCTCGTCGAATC |  |
| qPCR-Sspka2 F | CCGAACAGCTACAGCCGATTCTC |  |
| qPCR-Sspka2 R | TGCCAGAACAATTCACCTCCAGAC |  |
| qPCR-SsMkk1 F | CGAGCACATCTGTCGCTACTATGG |  |
| qPCR-SsMkk1 R | TGGAGATAGGTCAGACCGTTCAGG |  |
| qPCR-SsMk1 F | CAGAGGATGAACCTACCGCCAAC |  |
| qPCR-SsMk1 R | AGTTGTTCCTTGGTGAGGTTGTCC |  |
| qPCR-SsRgb1 F | GTTGGTGGTCAAGGTGGAAGGATG |  |
| qPCR-SsRgb1 R | GGTTGTTCGTTGCAGCAATCGC |  |
| qPCR-SsCna1 F | AAGCCGCTGTGTTGAAGTACGAG |  |
| qPCR-SsCna1 R | CCGAGGAAGTGCTGAGGATTGC |  |
| qPCR-SsPac1 F | CAACCTGGTGCTCACTACACTCAC |  |
| qPCR-SsPac1 R | TTGGTGCTGAAGCGTAAGAAGTGG |  |
| qPCR-SsEmp1 F | TATCACAAGCAGCAACAGCACCTC | Profiling infection cushions -related gene expression |
| qPCR-SsEmp1 R | AGCGGTGGTGGAGGTAGAAGTTC |  |
| qPCR-SsMst12 F | CGTCGATTCCACCTGGAGTTGC |  |
| qPCR-SsMst12 R | CTCCTCAGGTCCGACGGTCTATG |  |
| qPCR-Sspls1 F | AAGGCTGGTACTTCACAAGCACTG |  |
| qPCR-Sspls1 R | CGCAGACGACGACCATGTAGC |  |
| qPCR-SsChm1 F | CGGCTCAGCAACAACCATCTAGG |  |
| qPCR-SsChm1 R | GCGGCTGGCTTGGCAGTAAG |  |
| qPCR-SscpkA F | CGCTGCATCCTGTAGCTGGTAAC |  |
| qPCR-SscpkA R | CTGATCTGTGGCTGCTGCTGTG |  |
| qPCR-SsMas2 F | CGGTGTCAAGGCTGCTGAAGG |  |
| qPCR-SsMas2 R | GTGCTGTCAATCATTGCGGTCAAG |  |
| pCB-*Ssnsd1*-GFP F | CGGATCCATGGCTGCACCGATAAGT | SsNsd1- GFP fusion protein |
| pCB-*Ssnsd1*-GFP R | GGAATTCTGACCGTGGTGAATGAT |  |
| pNDB-*Ssfdh1*-mCherry F | CATGCCATGGGTTCTCTCATATTAACCACACACC | SsFdh1-mCherry fusion protein |
| pNDB-*Ssfdh1*-mCherry R | CATGCCATGGATGCCGGCTGATACTGTTG |  |
| pET28a-*Ssnsd1* F | GGAATTCATGGCTGCACCGATAA | Protein expression of SsNsd1 and SsFdh1 in *E.coli* |
| pET28a-*Ssnsd1* R | CGAGCTCTGACCGTGGTGAATGAT |  |
| pET28a-*Ssfdh1* F | GGAATTCATGCCGGCTGATACTGTTGG |  |
| pET28a-*Ssfdh1* R | CGGATCCTTCTCTCATATTAACCACACACC |  |
| Y2H-*Ssnsd1*-F | GCGGAATTCATGGCTGCACCGATAA | SsNsd1 and SsFdh1 Y2H |
| Y2H-*Ssnsd1*-R | AACTGCAGTGACCGTGGTGAATGAT |  |
| Y2H-*Ssfdh1*-F | GGAATTCATGCCGGCTGATACTGTTGGGAAG |  |
| Y2H-*Ssfdh1*-R | CGAGCTCGTTCTCTCATATTAACCACACACCTA |  |
| BiFC-*Ssnsd1* F | GCGGAATTCATGGCTGCACCGATAAGT | SsNsd1 and SsFdh1 BiFC |
| BiFC-*Ssnsd1* R | GCGTCGACTGACCGTGGTGAATGAT |  |
| BiFC-*Ssfdh1* F | CGAGCTCATGCCGGCTGATACTGTTGG |  |
| BiFC-*Ssfdh1* R | GCTCTAGATTCTCTCATATTAACCACACACC |  |
| CoIP-*Ssnsd1*-GFP F | CGGATCCATGGCTGCACCGATAAGT | SsNsd1 and SsFdh1 co-IP |
| CoIP-*Ssnsd1*-GFP R | CGCGGTCGACTGACCGTGGTGAATGAT |  |
| CoIP-*Ssfdh1*-3×FLAG F | GAATTCATGCCGGCTGATACTGTTGGGAAG |  |
| CoIP-*Ssfdh1*-3×FLAG R | GCGTCGACTTCTCTCATATTAACCACACACCTA |  |
| F1-L-F | CGGAATTCTATAGCGAGGTTCTACTGTATGTGT | *Ssfdh1* gene replacement KO |
| F1-L-R | CGAGCTCGTTTCCTATTCTCTTTTTCGTTC |  |
| F1-R-F | CGGGATCCCTGCATGGGGTTATAAGGGA |  |
| F1-R-R | GCTCTAGACACTTCGAACTAACAATAATTCG |  |
| F1-F | CGAATTCATGCCGGCTGATACTGTTGGGAAG | Verified knockout (KO) strains; Southern probe 2 |
| F1-R | GCGTCGACTTCTCTCATATTAACCACACACCTA |  |
| H1 | AACTGATATTGAAGGAGCATT |  |
| H2 | AACTGGTTCCCGGTCGGCATC |  |
| Hpt-a | GCGAAGAATCTCGTGCTTTC |  |
| Hpt-b | CGTCTGCTGCTCCATACAAG |  |
| C-F1-F | CCGCTCGAGTATAGCGAGGTTCTACTGTATGTGT | *Ssfdh1* genetic complement |
| C-F1-R | ATAAGAATGCCACTTCGAACTAACAATAATTCG |  |
| G4-F | GGTACCGATATGATTGAACAAGATG | Southern probe 3 |
| G4-R | GTCGACTCCTCAGAAGAACTCGTC |  |
| F1O-F | AACACTCCCCAGCCCACTACATTC | Southern probe 1 |
| F1O-R | CAACGGAAATATCGGCAACGACAGT |  |
| qPCR-Histone-F | GGCTCGTACCAAGCAAACTG | qPCR reference  gene |
| qPCR-Histone-R | GAAGTCTTGGGCGATTTCAC |  |
